# Numerical study of magnetic hyperthermia ablation of breast tumor on an anatomically realistic breast phantom

**DOI:** 10.1371/journal.pone.0274801

**Published:** 2022-09-21

**Authors:** Reza Rahpeima, Chao-An Lin

**Affiliations:** Department of Power Mechanical Engineering, National Tsing Hua University, Hsinchu, Taiwan; Tongji University, CHINA

## Abstract

Magnetic fluid hyperthermia (MFH) is a novel reliable technique with excellent potential for thermal therapies and treating breast tumours. This method involves injecting a magnetic nanofluid into the tumour and applying an external AC magnetic field to induce heat in the magnetic nanoparticles (MNPs) and raise the tumour temperature to ablation temperature ranges. Because of the complexity of considering and coupling all different physics involves in this phenomenon and also due to the intricacy of a thorough FEM numerical study, few FEM-based studies address the entire MFH process as similar to reality as possible. The current study investigates a FEM-based three-dimensional numerical simulation of MFH of breast tumours as a multi-physics problem. An anatomically realistic breast phantom (ARBP) is considered, some magnetic nanofluid is injected inside the tumour, and the diffusion phenomenon is simulated. Then, the amount of heat generated in the MNP-saturated tumour area due to an external AC magnetic field is simulated. In the end, the fraction of tumour tissue necrotized by this temperature rise is evaluated. The study’s results demonstrate that by injecting nanofluid and utilizing seven circular copper windings with each coil carrying 400 A current with a frequency of 400 kHz for generating the external AC magnetic field, the temperature in tumour tissue can be raised to a maximum of about 51.4°C, which leads to necrosis of entire tumour tissue after 30 minutes of electromagnetic field (EMF) exposure. This numerical platform can depict all four various physics involved in the MFH of breast tumours by numerically solving all different equation sets coupled together with high precision. Thus, the proposed model can be utilized by clinicians as a reliable tool for predicting and identifying the approximate amount of temperature rise and the necrotic fraction of breast tumour, which can be very useful to opt for the best MFH therapeutic procedure and conditions based on various patients. In future works, this numerical platform’s results should be compared with experimental *in-vivo* results to improve and modify this platform in order to be ready for clinical applications.

## 1. Introduction

Breast cancer is one of the most common cancers nowadays, and many people from both genders die because of this type of cancer every year. According to the American cancer society’s statistics, the estimated new breast cancer cases and deaths in 2021 are 284200 and 44130 people in the USA [1]. The use of heat as a tool for eliminating malignant tumours has been known since ancient times and various techniques have been developed in the last century. However, only in the past few decades, it has been used for ablation and treatment of tumours in a controlled procedure called hyperthermia [2, 3].

*Hyperthermia* is a heating procedure in which the tumour’s temperature rises above the normal body temperature (37°C) and is held for several minutes to damage and ablate cancerous tissues [4]. Heating temperatures between 41–46°C are considered as moderate hyperthermia, and between 46–56°C and higher, as thermal ablation range. These temperatures can cause tissues to undergo coagulation or tissue necrosis, called thermo-ablation [4]. Unlike other traditional cancer treatment procedures such as chemotherapy, radiotherapy, and surgery, hyperthermia is a non-invasive procedure that eliminates just cancerous tissues and minimally damages normal surrounding body tissues [5]. There are various approaches to conduct the hyperthermia process, such as microwaves, laser beams, high-intensity focused ultrasound (HIFU), or magnetic fluid hyperthermia (MFH) [5].

Magnetic fluid hyperthermia (MFH) is a treatment that involves injecting magnetic fluid into the tumour and then applying an external electromagnetic field. It can raise the temperature of the tumour to 43°C or more, causing apoptosis. In recent decades, researchers have considered the application of magnetic fluids in hyperthermia treatments. Because, unlike laser, microwave, and ultrasound hyperthermia, this minimally invasive treatment avoids overheating healthy tissues. In this method, the magnetic field is absorbed solely by magnetic nanoparticles (ingredients of magnetic fluids). Also, unlike other noninvasive treatment techniques, this method can be used to treat deep-seated tumours [6, 7]. Many researchers have investigated the MFH technique. Nevertheless, there is still a lack of complete numerical study that can predict this complicated multiphysics phenomenon on realistic phantoms. Some of the most recent and prominent approaches in this field are reviewed below. Since the current research aims to study this phenomenon numerically, it is tried to mostly focus on analytical and numerical studies in this field.

In 2010, Miaskowski et al. [8] proposed a lump system approach for determining the temperature increase in breast cancer based on the specific absorption rate by utilizing magnetic nanoparticles (MNPs). This formula matches the results of experiments on female breast cancer phantoms. To gain further insight into the temperature distribution, Miaskowski et al. [6] performed a numerical study of MFH to treat breast cancer. An artificial breast phantom was created, and the simulation results were compared with the experimental measurements on this phantom. Further, Attar et al. [9] carried out *in-vitro* experimental research on MFH in soft tissue by considering artificial blood perfusion. The focus was to investigate the thermal behaviour of dead kidney tissue during MFH. Recently, Suleman et al. [10] carried out a numerical simulation of MFH of breast tumours using magnetite (Fe_3_O_4_) nanoparticles. A 3D FEM (finite element method) based model investigated different physics incorporated in the MFH process. By adopting a realistic breast model, Miaskowski and Subramanian [11] performed a calorimetric study of MFH treatment of breast cancer. They used the magnetic-field-dependent Néel and Brownian relaxation times to compare the interactions of MNPs and tumour tissue. Gas et al. [12] carried out an *in-silico* study on tumor-size-dependent thermal profiles inside an anthropomorphic female breast phantom. They exposed the breast tissues to an EMF generated by an eight-element dipole antenna matrix surrounding it and used a finite-difference time-domain (FDTD) engine for solving the Maxwell equations coupled with the modified Pennes’ bioheat equation. Raouf et al. [13] carried out a parametric numerical investigation of MFH using finite element analysis. They predicted temperature distribution during the MFH process. Suleman et al. [14] introduced a mathematical modeling approach toward MFH of cancer. In their research, they described the basic physical mechanisms behind this treatment modality and introduced recent advances in the mathematical modeling approach toward this therapy. Also, many researchers simulate breast tissue thermal responses by various mathematical models. Neto et al. [15] produced a simplified 3D mathematical model to predict the human breast thermal response directly from physical laws and making use of mass, heat, and fluid flow empirical and theoretical correlations. Gas et al. [16] carried out a FEM-based analysis of temperature in the anatomical model of the female breast with a strictly defined level of power generated by the EMF source in pathological tissue saturated with ferrofluid. The nanofluid injection method can affect the MFH process as well. Gas et al. [17] investigated the influence of multi-tine electrode configuration in realistic hepatic radiofrequency ablative heating. They utilized the Arrhenius model to establish the thermal damage of hepatic tissue. Tang et al. [18] investigated the effect of injection strategy for nanofluid transport on thermal damage behavior inside biological tissue during magnetic hyperthermia. Rajput et al. [19] carried out a computational feasibility study to model heat flow for a controlled focusing of microwave hyperthermia of breast cancer. Their study includes a mathematical analysis of hyperthermia and FEM modeling results. Ling et al. [20] introduced a microstrip antenna with different applied frequencies as a non-invasive hyperthermia applicator to clarify the sufficient heat distribution on the treated tissue for different breast cancer stages. They analyzed 57 mammogram breast cancer images from the early stage to stage 3 to obtain the required penetration depth and focus position distance. Nizam-Uddin et al. [21] established a simplified experimental setup to investigate the rise in temperature in a microwave hyperthermia treatment system for breast tumors. Their proposed system is illustrated by numerical simulations of breast phantom as well to investigate energy propagation in tissue layers at various excitation frequencies.

Based on the literature mentioned above, only a few studies have addressed challenges involving 3D modeling of MFH processes that can consider the entire governing equations of this phenomenon. Because of the complexity of a thorough FEM study, almost none of them considers an anatomically realistic breast phantom for this purpose. Therefore, to bridge this gap, a FEM-based simulation is carried out in the current study, which can address the entire MFH process problem and demonstrate the real physics involved in this phenomenon in great detail. Unlike previous studies, all different physics involved in the MFH procedure is considered and numerically simulated. Furthermore, by utilizing an anatomically realistic breast phantom (ARBP), the goal was to get outcomes that were as similar to reality as feasible. This ARBP was derived using one of the models in a collection of T1-weighted magnetic resonance imaging (MRIs) of patients in the prone position as explained in the next section [22].

The whole MFH process and the steps of the simulations used to predict reality using an ARBP are described here. First, diffusion of MNPs inside tumour tissue is simulated, and the concentration of these MNPs is obtained in different parts of the tumour and breast. After that, the AC magnetic field generation is simulated, and by calculating the amount of magnetic energy absorbed by the MNPs, the amount of heat-induced and temperature increase in the tumour area is calculated. By knowing the amount of temperature increase, the fraction of necrotic tissue is calculated. This thorough numerical platform can be utilized by clinicians as a reliable predicting tool for opting for the best therapeutic plan based on various patients.

The remainder of this paper is organized as follows: In section 2, the materials and methods are presented. In section 3, numerical results of the MFH processes are presented. Section 4 provides a conclusion for the study.

## 2. Material and methods

This section begins with a brief overview of the MFH procedure. Then there is a description of governing equations and mathematical models. The remaining subsections provide essential information about various stages of the numerical modelling process.

### 2.1 MFH process

The MFH aims to induce local heating by utilizing some radio-frequency electromagnetic waves. For this purpose, first, MNPs should be injected inside the tumour. Due to the diffusion process, these MNPs will propagate inside the tumour and its surroundings. After that, generating an external AC magnetic field in the breast area would induce heat in these MNPs. Through the magnetic coupling between the magnetic component of the generated magnetic field and the magnetic moment of MNPs, MNPs can be employed as nano-heaters triggered by an external AC magnetic field. The energy absorbed by these MNPs is dissipated as heat by this coupling mechanism [23].

Dynamic hysteresis losses caused by the relaxation of the magnetic moments generate this heat induction in MNPS. The relaxation process consists of two simultaneous mechanisms. One of them is called Brownian relaxation and is correlated to the physical rotation of the MNPs on the surrounding medium, which depends on the medium viscosity and hydrodynamic volume of MNPs. The other one is the Neel relaxation, which relates to the rotation of the atomic magnetic moments within the crystal lattice of the MNPs. Both mechanisms coincide for some frequency range; however, they are independent. The faster mechanism is the dominant one for heat induction [24].

This heat induction leads to a temperature increase in the surrounding medium (tumor). If the temperature remains high (over 41°C) for some time, tumour tissues will start to be damaged and die, which is called Thermal ablation. So, different steps of MFH can be outlined as follows:

1. Injection of MNPS and diffusion of them inside a tumour and its surrounding.
2. Generating an external magnetic field and inducing heat in the MNPs.
3. Temperature increment in the tumour leads to necrosis of the tissues.

**Fig 1:**
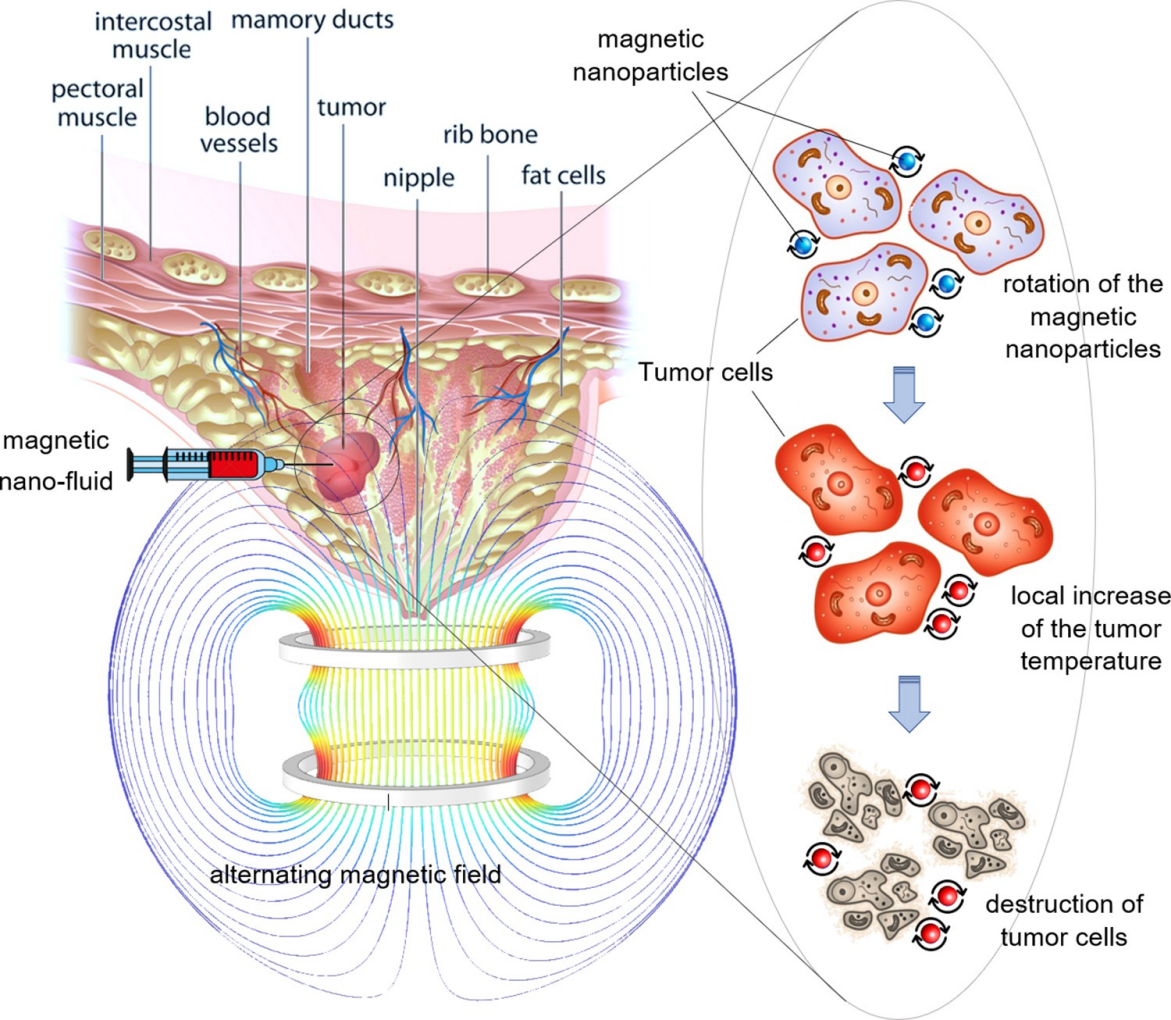
A schematic view of the MFH process of a breast tumor.

For a better understanding, a schematic of the MFH process on a breast tumour is illustrated in Fig 1.

### 2.2 Governing equations

The governing equations that should be solved for a thorough simulation of the MFH process include four parts. Diffusion of MNPs in the tumour, generation of the magnetic field by a current-carrying coil, heat generation and heat transfer in biological tissue, and prediction of the fraction of the tumour damage and necrosis. The governing equations for each of these steps are mentioned in the following.

#### 2.2.1 Diffusion of MNPs. According to the Fick’s second law, the considered diffusion equation is as follows [10, 25]:


$$\frac{\partial c}{\partial t}+D\nabla^{2}c=0$$ (1)

$$D=\frac{k_{B}T}{6\pi \eta r_{p}}$$ (2)

In the above equations, *c* is the concentration of the MNPs, and *D* is the diffusion coefficient formulated by the Stokes-Einstein equation and considered to be constant for the given fluid. Also, k_B_, *T*, *η*, and *r*_p_ are Boltzmann constant (1.38×10^−23^ J K^-1^), absolute temperature, nanofluids viscosity, and radius of the MNPs, respectively.

#### 2.2.2 Magnetic field generation and power losses dissipated by MNPs

The governing equations for a magnetic field generated by a current-carrying coil in the frequency-transient domain can be expressed by the Maxwell-Ampere’s and Faraday’s laws as follows [6, 26]:

$$\nabla \times \vec{H}=\vec{J}+\frac{\partial \vec{D_{e}}}{\partial t}$$ (3)

$$\nabla \times \vec{E}=-\frac{\partial \vec{B}}{\partial t}$$ (4)

$$\vec{D_{e}}=\epsilon_{0}\epsilon_{r}\vec{E}$$ (5)

$$\vec{B}=\mu_{0}\mu_{r}\vec{H}$$ (6)

$$\vec{J}=\sigma \vec{E}$$ (7)

In the above equations, $\vec{H}$ is the vector of magnetic field strength, $\vec{B}$ is the vector of magnetic flux density, $\vec{E}$ is the vector of electric field strength, $\vec{D_{e}}$ is the vector of electric flux density or electric displacement, $\vec{J}$ is the vector of current density, ϵ_0_ is the permittivity of free space (8.85×10^−12^ F m^-1^), *ϵ*_r_ is relative permittivity, μ_0_ is the permeability of free space (4π×10^−7^ H m^-1^), *μ*_r_ is relative permeability, and *σ* is electrical conductivity. It should be mentioned that the current density inside the homogenized multi-turn source coil can be expressed as follows [27]:

$$J_{coil}=\frac{NI_{coil}}{A_{coil}}$$ (8)

In this equation, *N*, *I*_coil_, and *A*_coil_ are the number of turns, coil current, and total cross-section of the coil domain, respectively.

In the presence of this external alternating magnetic field (AMF), the amount of power losses dissipated by MNPs can be modeled by the Rosensweig formulation as follows [6, 28]:

$$P_{dis}=\pi \mu_{0}\chi_{0}H_{0}^{2}f\frac{2\pi f\tau }{1+{\left(2\pi f\tau \right)}^{2}}$$ (9)

$$\chi_{0}=\mu_{0}M_{s}^{2}V_{M}/k_{B}T$$ (10)

In the above equations, μ_0_ is the permeability of free space equal to 4π×10^−7^ H m^-1^, *χ*_0_ is ferrofluid susceptibility, *H*_0_ is the amplitude of magnetic field strength, *f* is the cyclic frequency equal to *ω*/2π, *ω* is the angular frequency, *τ* is relaxation time, *M*_s_ is the magnetic fluid saturation magnetization, *V*_M_ is the magnetic volume of MNP equal to 4π*r*_p_^3^/3 for a nanoparticle with a radius equal to *r*_p_, and *T* is the absolute temperature. It should be mentioned that in this formula, ferrofluid susceptibility (*χ*_0_) is assumed to be constant and not dependent on the magnetic field [6]. A comprehensive explanation of the derivation of these formulas and different parameters can be found in Rosensweig’s paper [28]. The effective relaxation time *τ* can be expressed as follows:

$$\frac{1}{\tau }=\frac{1}{\tau_{B}}+\frac{1}{\tau_{N}}$$ (11)

In this formula, *τ*_B_ and *τ*_N_ are Brownian and Neel relaxation times. As mentioned before there are two physical processes responsible for the dissipation of power and heat induction, that are Brownian and Neel relaxations, and can be expressed as follows [6, 28]:

$$\tau_{N}=10^{-9}\times e^{KV_{M}/k_{B}T}$$ (12)

$$\tau_{B}=3\eta V_{H}/k_{B}T$$ (13)

$$V_{H}={\left(1+\delta /R\right)}^{3}V_{M}$$ (14)

In th*e* above equations, *K* is the magnetic anisotropy constant, *η* is the viscosity of the magnetic fluid medium, *V*_H_ is the hydrodynamic volume of an MNP, and *δ* is the surfactant layer thickness.

#### 2.2.3 Heat transfer in biological tissues

Temperature distribution in biological tissues can be expressed by the Penne’s bio-heat transfer equation as follows [29]:

$$\rho c_{p}\frac{\partial T}{\partial t}=k\nabla^{2}T+\omega_{b}\rho_{b}c_{p,b}(T_{b}-T)+Q_{met}+Q_{MNPs}$$ (15)

In the above equation, *ρ* is the tissue density, *c*_p_ is the specific heat in constant pressure of the tissue, *k* is the thermal conductivity of the tissue, *ω*_b_ is local blood perfusion rate, *ρ*_b_ is the density of blood, *c*_p,b_ is the specific heat of blood, *T*_b_ is local arterial blood temperature, *Q*_met_ is local metabolic heat generation rate, and *Q*_MNPs_ is the heat dissipation rate by MNPs equal to *P*_dis_ mentioned in the previous section.

#### 2.2.4 Prediction of the fraction of necrotic tissue

The degree of tissue injury due to the hyperthermia process can be evaluated by the Arrhenius kinetic model as follows [17, 30–32]:

$$\frac{\partial \alpha }{\partial t}={\left(1-\alpha \right)}^{n}Ae^{\frac{-\Delta E}{RT}}$$ (16)

In this equation, *α* is the degree of tissue injury, *n* is the polynomial order of the equation (in this study *n* is considered to be 1), R is the universal gas constant (8.314 J K^−1^ mol^−1^), and *T* is the tissue temperature. Parameters *A* and Δ*E* are frequency factor and activation energy and are dependent on the type of tissue and have been characterized for different tissue types. These parameters for the breast tissue are calculated to be *A* = 1.18×10^44^ s^-1^ and Δ*E* = 3.02×10^5^ J mol^-1^ [33]. After calculating the degree of tissue injury, the fraction of necrotic tissue (*θ*_d_) is expressed by [31, 32]:

$$\theta_{d}=min(\max\left(\alpha ,0\right),1)$$ (17)

### 2.3 Description of the model’s geometry

**Fig 2:**
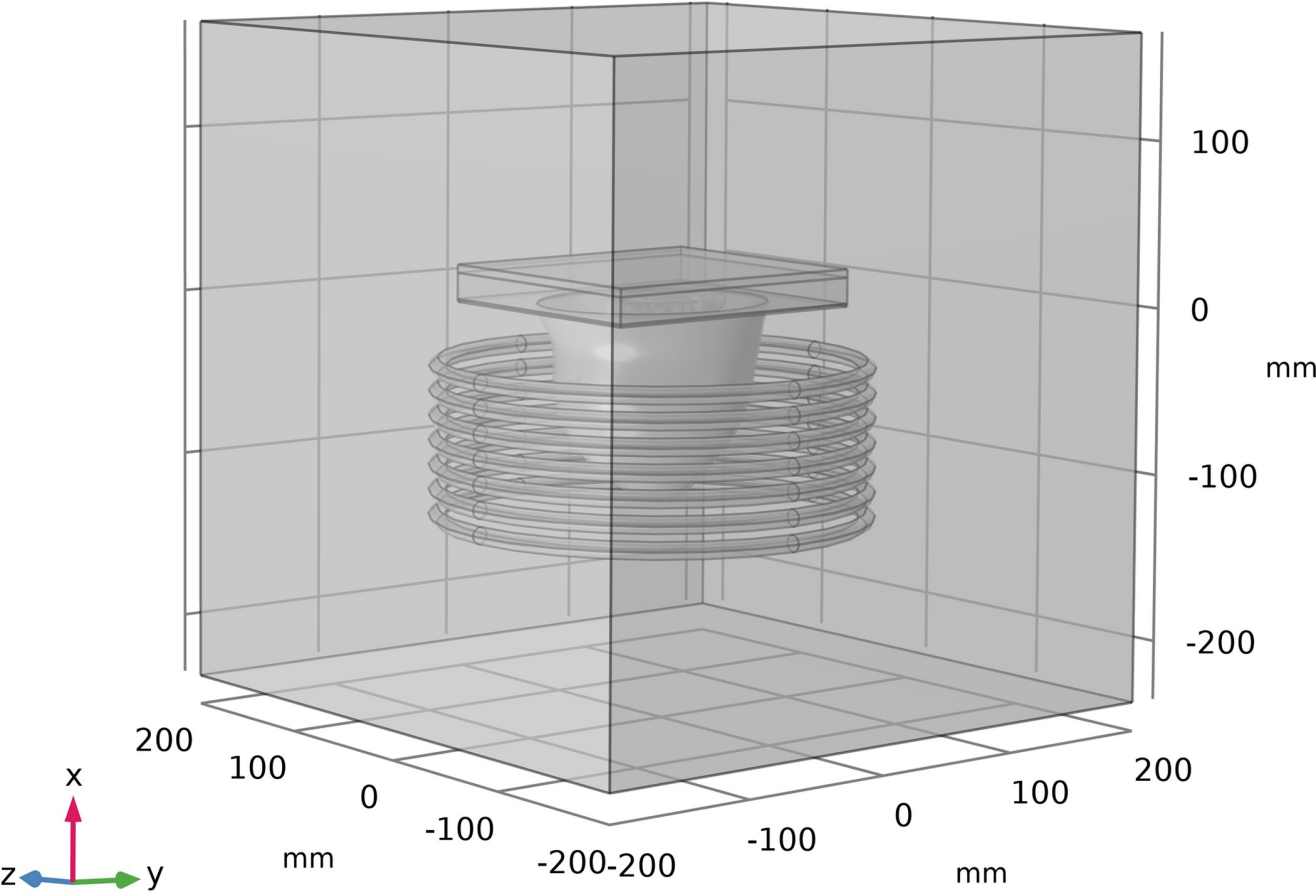
3D presentation of the whole considered model geometry.

The FEM-based commercial software COMSOL Multiphysics [34] is used to carry out all of the simulations in this paper. The 3D cartesian coordinate system is utilized and as mentioned before, an ARBP is utilized and located inside of a current-carrying coil. This coil consists of seven turns located vertically with a free space in between each of them. This considered coil generates a magnetic field in the breast area. The diameter of the coil and the distance between the coil windings are considered to be 260 mm and 15 mm, respectively, so the breast could locate inside of it completely. Also, the cross-section of coil wire is considered to be 1e-6 m^2^. To implement the magnetic field around the breast, all of these parts are located inside a hypothetical cube filled with air. This whole considered geometry is shown in Fig 2.

The following are descriptions of the steps involved in importing and applying 3D grid-based anatomically realistic numerical breast phantoms in the COMSOL software. The numerical breast phantom used in our simulations was chosen from the numerical breast phantom repository created by UWCEM (University of Wisconsin cross-disciplinary electromagnetics laboratory) [35], which includes several anatomically-realistic MRI-derived numerical breast phantoms for breast cancer detection. These breast phantoms were made using a series of T1-weighted MRI scans taken from individuals lying in a prone posture. Short TE (time to echo) and TR (time of repetition) durations are used to create T1-weighted pictures. The time interval between successive pulse sequences applied to the same slice is referred to as TR. The time between the delivery of the RF (radio frequency) pulse and the receiving of the echo signal is called TE. The numerical breast phantom picked is from class 2 and has the number of Breast ID 012204. The American College of Radiology describes the categorization numbers concerning their radiological density as follows [36]; Class 1, nearly totally fat (less than 25% glandular tissue); class 2, dispersed fibro-connective/glandular tissue (between 25% and 50% glandular tissue); class 3, heterogeneously dense breast (between 51% and 75% glandular tissue); and class 4, highly dense breast (above 75% glandular tissue).

**Fig 3:**
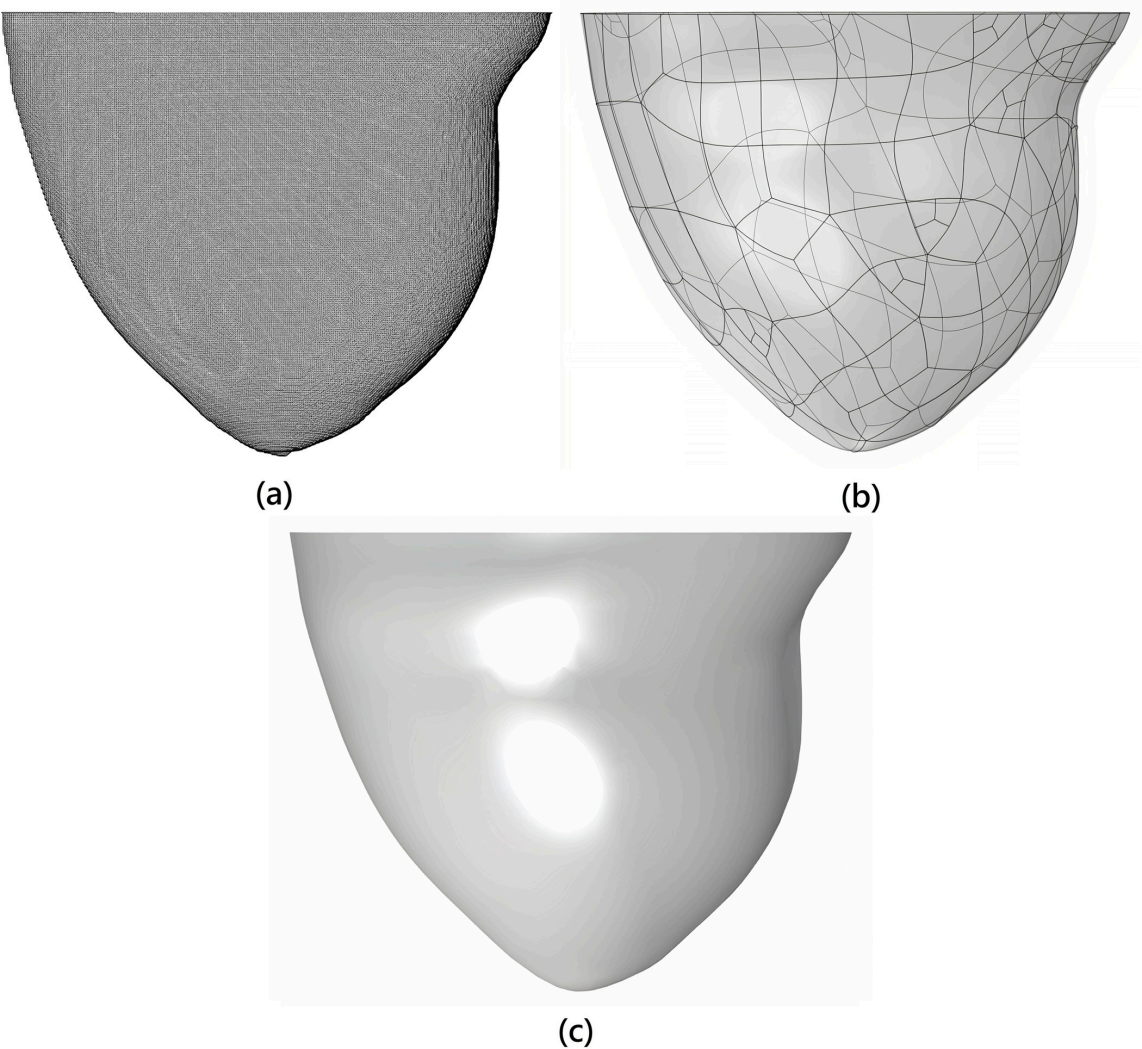
Different steps of creating a 3D breast phantom: (a) point cloud obtained from the database, (b) surfaces created from the point cloud, and (c) the volume created from knitted surfaces.

A cloud of points is created in 3D space using these mentioned data and a MATLAB [37] function that ties each point in the space to a specific tissue type of the breast phantom. The breast phantom is built as a 3D model by obtaining this point cloud for each tissue type and converting it to surfaces and volumes using CATIA software [38]. Several marginal points of each tissue type that could be roughly considered on one single surface were connected and produced surfaces (converting the point cloud to the surfaces). Many surfaces produced in this way were knitted together to produce an enclosed volume that represents each of the breast phantom’s tissue types (converting the surfaces to the volume). Five different tissue types are extracted to achieve a simplified ARBP, and data relevant to these tissue types are retrieved and used from the given database. The obtained and considered breast tissue types are (1) fibro-connective/glandular tissue (FCG), (2) transitional tissue, (3) fatty tissue, (4) muscle, and (5) skin. Views of the point cloud obtained from the specified database, the surfaces formed from this point cloud, and the volume developed from these surfaces relating to fatty tissue, as an example, are presented in Fig 3 to understand better the many processes of constructing a 3D breast phantom. Each of the tissue types mentioned is developed into a 3D object according to the stages elaborated in Fig 3.

**Fig 4:**
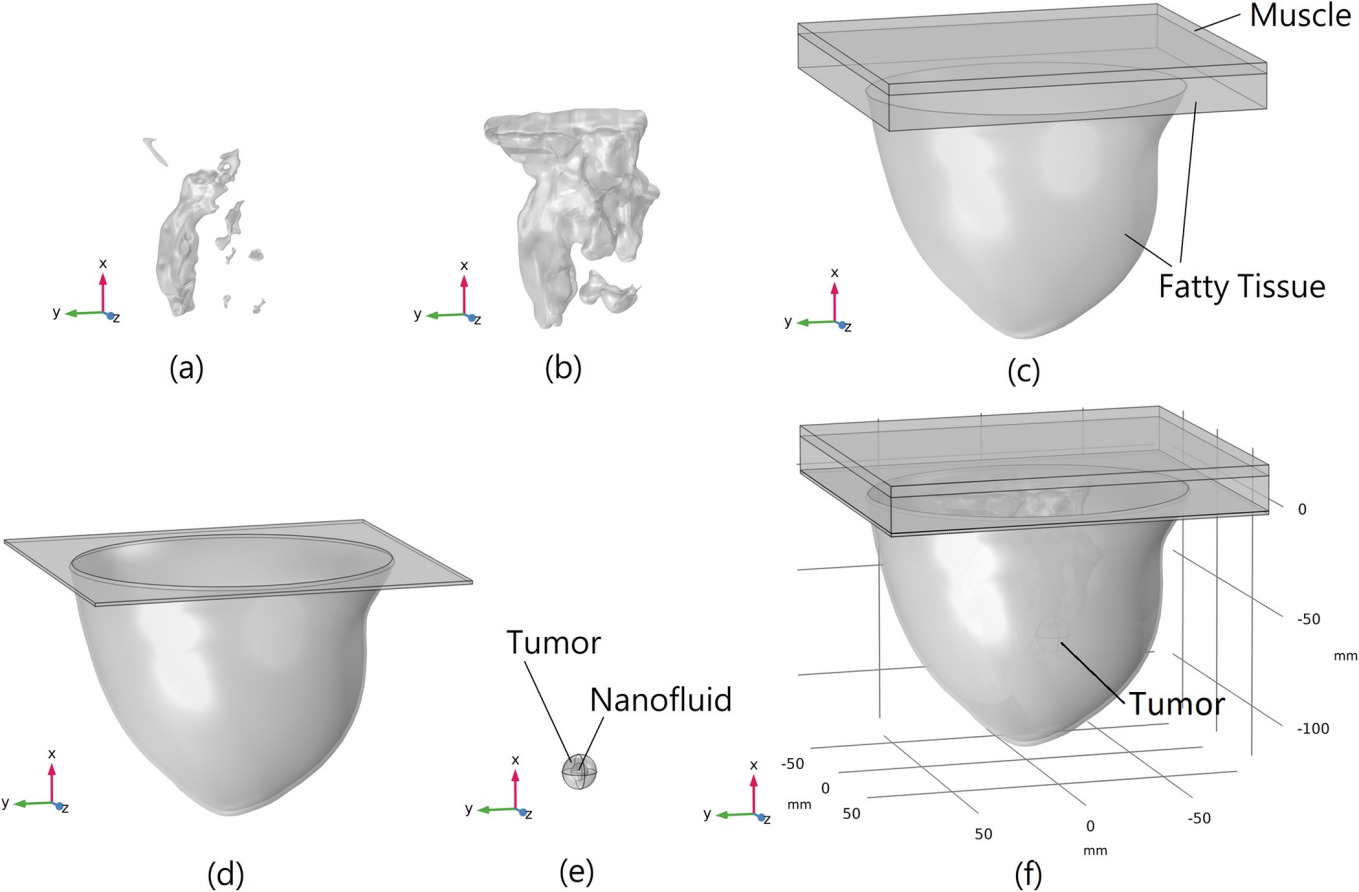
3D geometry of different tissue types separately and all together, (a) FCG tissue, (b) transitional tissue, (c) fatty tissue and muscle, (d) skin, (e) tumor and injected nanofluid, and (f) all of the tissues inside of each other as the ARBP.

The tumour in our simulations is considered as a sphere with a diameter equal to 16 mm and its center located at the point: *x* = −70 mm, *y* = −10 mm, and *z* = 10 mm. This tumour size is categorized as T1c [39] and is a small tumour in the early stages of cancer development. Inside the tumour, another sphere is considered with a diameter equal to 8 mm, representing the injected nanofluid inside the tumour. Right after the injection, the nanofluid is located as a sphere within the tumour, and through time, it diffuses outward to the other tumour and breast areas. Also, it should be mentioned that this tumour is located inside the fatty tissue of the breast phantom. In Fig 4, all of the tissue types mentioned along with the tumour are shown separately and all together as the ARBP.

### 2.4 Numerical methodology and simulation procedure

In this section, the numerical modelling of MFH will be introduced step by step. This section is divided into several subsections and each of these steps is explained accordingly in these subsections. The needed information, material properties, and initial and boundary conditions for each physics will be explained. First, the simulation of diffusion of MNPs inside the tumour and breast tissues should be discussed.

#### 2.4.1 Diffusion simulation

The nanofluid considered in the current study is a diluted fluid consisting of magnetite MNPs with the base fluid water. The volume fraction of MNPs in the fluid (shown in Eq 18) is considered to be 0.2. These assumptions are considered based on similar papers [6, 10].


$$VF=\frac{V_{np}}{V_{nf}}$$ (18)

In the above equation, VF, *V*_np_ and *V*_nf_ are volume fraction of MNPs in the nanofluid, total volume of nanoparticles, and volume of nanofluid. According to previous clinical and non-clinical studies, different dosages of nanofluid injection can be considered based on the tumor size and the time considered for each MFH therapy session. In the current study, the total volume of nanofluid injection inside the tumor is considered to be equal to 0.1 mL, which is less than half of the amount considered by Tang et al. [40] (They considered a total volume of 0.24 mL nanofluid injection). Thus, this volume of nanofluid can be expressed as a safe amount. By having VF and *V*_nf_, *V*_np_ will be calculated as 0.02 mL. By having magnetite density and molar mass based on Table 1. The amount of MNPs moles injected can be calculated as 4.47×10^−4^. By considering that all of the injected nanofluid will be accumulated inside of a sphere with an 8 mm diameter at the beginning, the initial concentration of MNPs in this sphere will be calculated as 1669 mol m^−3^. Transport of diluted species physic is considered in COMSOL software and diffusion of these MNPs is simulated inside of the tumor and breast porous media. A time-dependent study is considered and the diffusion process is considered to be equal to 24 hours. It means that after injection of nanofluid, one day will be given to the nanofluid to diffuse completely inside the tumor area. So, after 1 day of injection, the patient will come for the MFH therapy. By considering the diameter of MNPS equal to 19 nm and based on Eq 2, the diffusion coefficient can be calculated as 5.01×10^−11^ m^2^ s^−1^. The concentration distribution of the MNPs in the tissue will be simulated in this step.

**Table 1:** Properties of different materials used for frequency of 400 kHz [6, 10, 45, 49–55].

Materials	*ρ* (kg m^-3^)	*C*_p_ (J kg^-1^ K^-1^)	*k* (W m^-1^ K^-1^)	*ϵ*_*r*_	*σ* (S m^-1^)	*Q*_met_(W m^-3^)	*ω*_b_(s^-1^)
**FCG tissue**	1050	3770	0.48	3.01e+3	5.4e-1	700	0.0067
**Transitional tissue**	990	3270	0.345	1.53e+3	2.83e-1	700	0.0067
**Fatty tissue**	930	2770	0.21	5.47e+1	2.51e-2	700	0.0067
**Tumor**	1050	3852	0.54	7.11e+3	3.73e-1	5790	0.005
**Muscle**	1100	3800	0.48	7.11e+3	3.73e-1	5790	0.005
**Skin**	1109	3391	0.37	1.11e+3	7.15e-4	400	0.0083
**Blood**	1050	3617	0.52	5.03e+3	7.06e-1	_	**_**
**MNPs**	5180	670	9.7	1.2e+1	2.5e+4	_	**_**
**Nanofluid**	1838	2201	1.2	3.75e+1	7.7e+3	_	**_**
**Air**	1.18	1005	0.026	1	5e-15	_	**_**
**Copper**	8960	385	400	1	5.99e+7	_	**_**

Diffusion of MNPs inside tumour and breast tissue will change the effective thermal and dielectric properties of tissue. These effective properties can be calculated based on the amount of volume fraction of MNPs in tissue. Effective properties of ferrofluid saturated tissues can be calculated as follows [6, 41–43]:

$$\theta =\frac{V_{np}}{V_{tissue}}$$ (19)

$$\rho_{ef}=(1-\theta )\rho_{tissue}+\theta \rho_{MNPs}$$ (20)

$$c_{p,ef}=(1-\theta )c_{p,tissue}+\theta c_{p,MNPs}$$ (21)

$$\frac{1}{k_{ef}}=\frac{\left(1-\theta \right)}{k_{tissue}}+\frac{\theta }{k_{MNPs}}$$ (22)

$$\frac{1}{\sigma_{ef}}=\frac{\left(1-\theta \right)}{\sigma_{tissue}}+\frac{\theta }{\sigma_{MNPs}}$$ (23)

In the above equations, *θ*, *V*, *ρ*, *c*_p_, *k*, and *σ* are volume fraction of MNPs in tissue, volume, density, specific heat in constant pressure, thermal conductivity, and electrical conductivity, respectively. It should be emphasized that *θ* is different from the VF mentioned previously. *θ* is the volume fraction of MNPs in whole tissue while VF is the volume fraction of MNPs in just nanofluid. The concentration of the MNPs in tissue can easily be converted into the volume fraction of MNPs in tissue by the use MNPs density and molar mass. Therefore, by using the concentration distribution of the MNPs obtained, all of the effective thermal and dielectric properties of tissue in each point of the breast tissue will be obtained according to the above equations. These obtained effective properties will be utilized in the next steps of computer simulation.

#### 2.4.2 Magnetic field simulation

After diffusion simulation, a magnetic field should be generated, and the amount of heat and the fraction of necrotized tissue due to this heat rise should be simulated. For this purpose, two physics, magnetic fields and bioheat transfer, should be coupled together. For the magnetic field generation, seven circular copper coils considered that each coil carries 400 A current. A transient frequency analysis is considered for 30 minutes of the MFH process, and the frequency is considered to be 400 kHz. The generated magnetic field will be obtained in this step.

#### 2.4.3 Bioheat transfer simulation

Bioheat transfer physic considers free heat convection and radiation to the ambient air at the breast’s skin. Also, blood perfusion has a significant impact on cooling the breast and tumour area. So, it is also considered (as seen in Eq 15). The properties of blood and blood perfusion rate and the metabolic heat source for each of the tissue types are mentioned in Table 1. Thermal damage is added, and the Arrhenius kinetics formula (Eq 16) calculates the fraction of necrotic tumours. In this step, the amount of induced heat inside tissue and the fraction of necrotized tissue will be simulated.

#### 2.4.4 Boundary conditions

**Fig 5:**
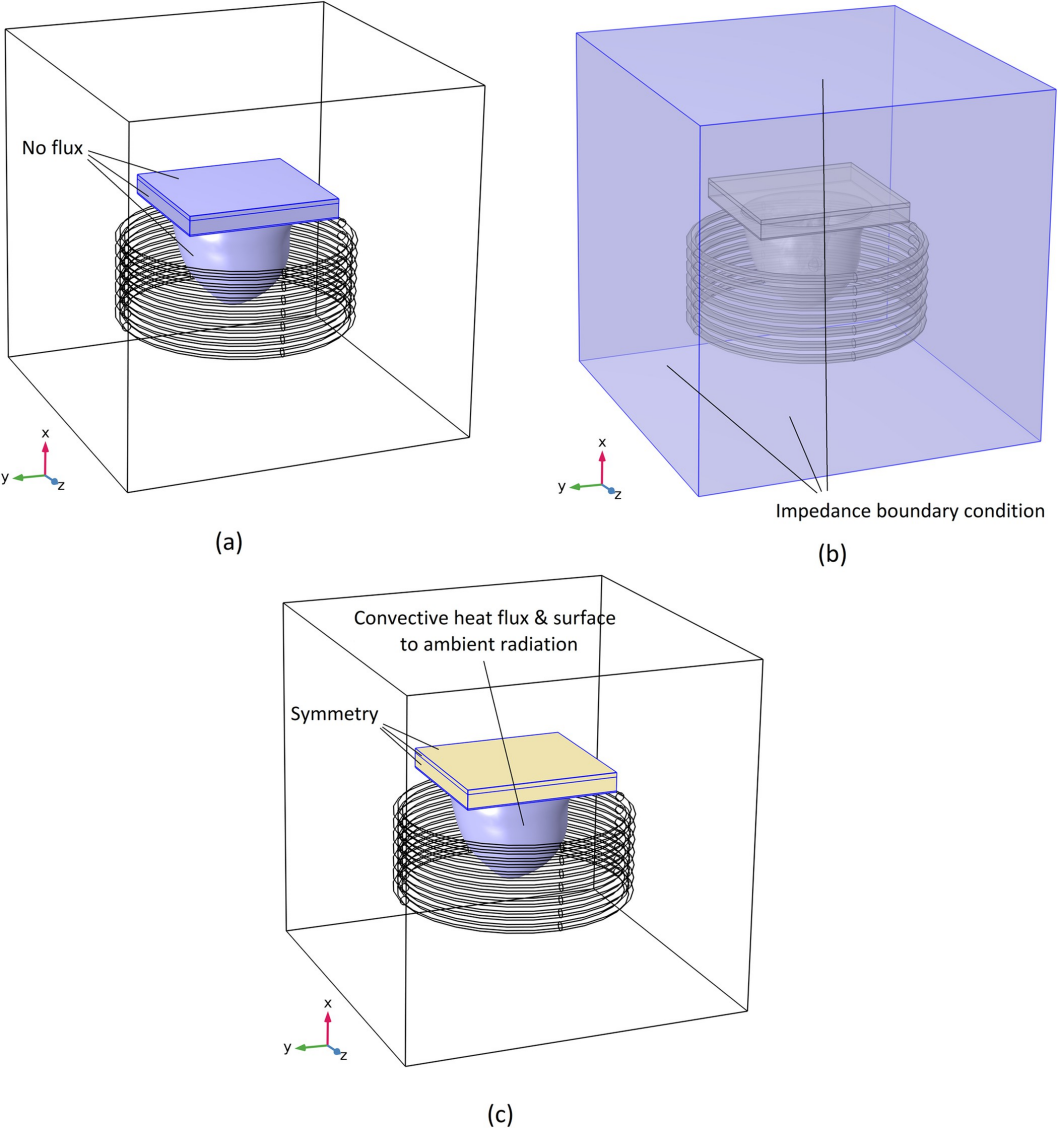
Boundary conditions considered in different physics, (a) transport of diluted species, (b) magnetic fields, and (c) bioheat transfer.

Boundary conditions considered for each of these mentioned three physics are shown in Fig 5.

As seen in this figure, for the transport of diluted species physics, the boundary condition for all outer boundaries of the breast phantom is considered no flux. For the magnetic fields physic, the boundary condition for every side of the hypothetical tube is assumed to be the impedance boundary condition, and air properties are defined as the material properties needed for these boundaries. For the bioheat transfer physic, both heat flux and surface to ambient radiation are considered for the skin boundaries to apply the free heat convection and radiation to the ambient air. Also, the symmetry boundary condition is considered for other boundaries except for the skin. Since these boundaries are located inside the body, it is considered that the heat flux across the boundary is zero, and the most similar boundary to reality is the symmetry boundary. The formulation for all these boundary conditions mentioned are as follows [44]:

$$No\;flux:-\vec{n}∙{\vec{\varphi }}_{i}=0$$ (24)

$$Impedance\;boundary\;condition:\sqrt{\frac{\mu_{0}\mu_{r}}{\epsilon_{0}\epsilon_{r}-j\sigma /\omega }}\vec{n}\times \vec{H}+\vec{E}-(\vec{n}∙\vec{E})\vec{n}=(\vec{n}∙\vec{E_{s}})\vec{n}-\vec{E_{s}}$$ (25)

$$Convective\;heat\;flux:-n∙\vec{q}=h(T_{\infty }-T)$$ (26)

$$Surface\;to\;ambient\;radiation:-n∙\vec{q}=\varepsilon \sigma_{SB}(T_{\infty }^{4}-T^{4})$$ (27)

$$Symmetry:-n∙\vec{q}=0$$ (28)

In the above equations, $\vec{n}$, ${\vec{\varphi }}_{i}$, μ_0_, *μ*_r_, ϵ_0_, *ϵ*_r_, j, *σ*, *ω*, $\vec{H}$, $\vec{E}$, $\vec{E_{s}}$, $\vec{q}$, *h*, *T*_∞_, *T*, *ε* and σ_SB_ are surface normal vector, diffusive flux vector of component *i*, the permeability of free space, relative permeability, the permittivity of free space, relative permittivity, imaginary unit, electrical conductivity, angular frequency, vector of magnetic field, vector of electric field, vector of the electric field on the surface, vector of heat transfer, convection heat transfer coefficient, ambient temperature, temperature, surface emissivity, and Stefan-Boltzmann constant (5.67×10^−8^ W m^-2^ K^-4^), respectively.

#### 2.4.5 Material properties

All different material properties considered and utilized in the current study are as follows. the heat convection coefficient is considered to be 5 W m^-2^ K^-1^ for free heat convection of breast skin with the ambient air [45, 46]. The ambient temperature is considered to be 293.15 K and the surface emissivity of the skin is considered equal to 0.98 [47]. All of the utilized properties of different materials considered in the current study are mentioned in Table 1. Nanofluid’s effective properties can be calculated according to the formulas mentioned in reference [48] and mentioned in Table 1 as well.

In the above table, *ρ*, *C*_p_, *k*, *ϵ*_r_, *σ*, *Q*_met_, and *ω*_b_ are density, specific heat in constant pressure, heat conductivity, relative permittivity, electrical conductivity, metabolic heat generation rate, and blood perfusion rate respectively.

It should be mentioned that the discretization methods used in our simulations are linear for the Transport of diluted species physic, quadratic for the magnetic fields physic, and quadratic Lagrange for the bioheat transfer physic. All simulations are performed by a system equipped with an Intel Core i5-8600 Processor at 3.10 GHz and 32 GB DDR4 RAM, and the computation time for each simulation is approximately 27 hours.

**Fig 6:**
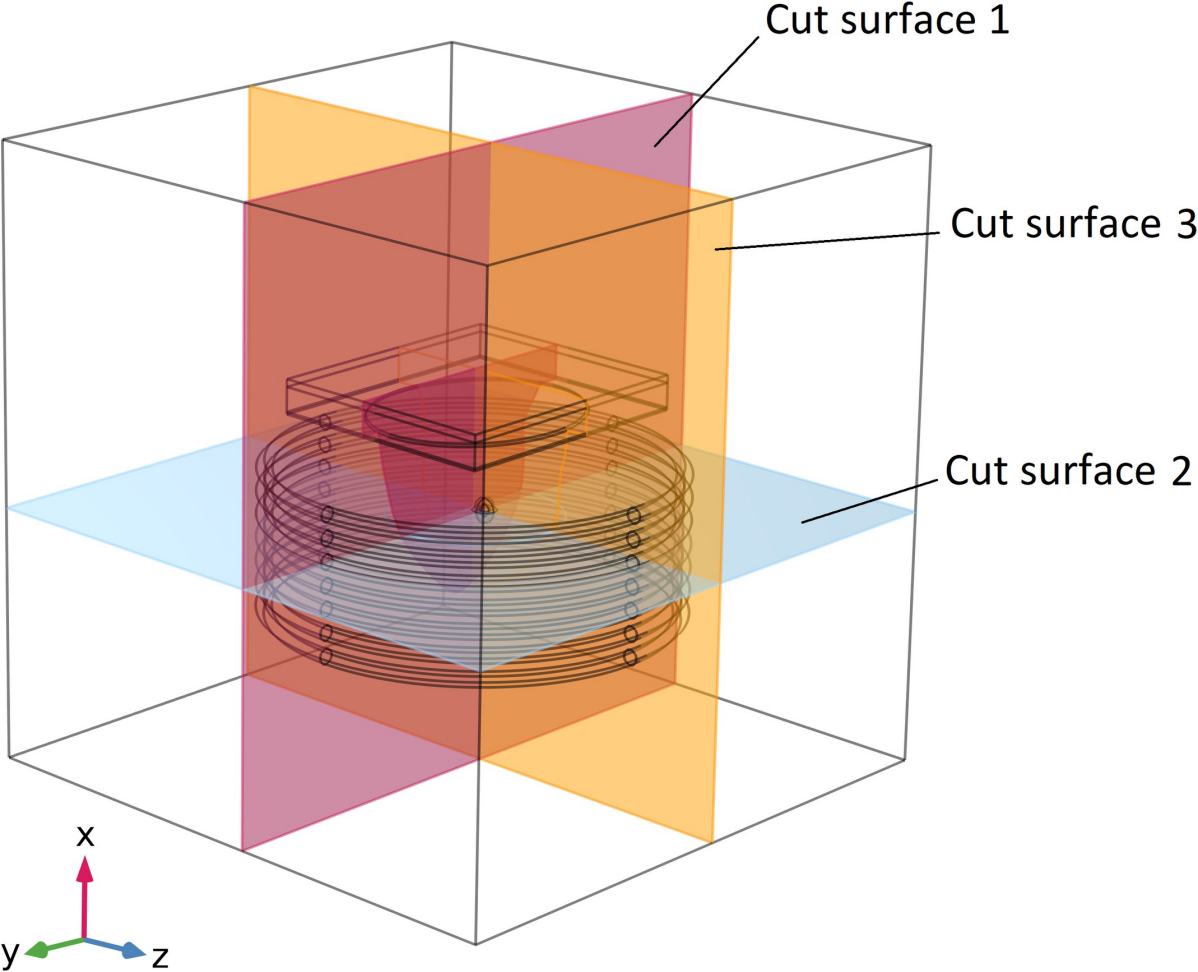
Considered cut surfaces that pass through the center of the tumor.

**Fig 7:**
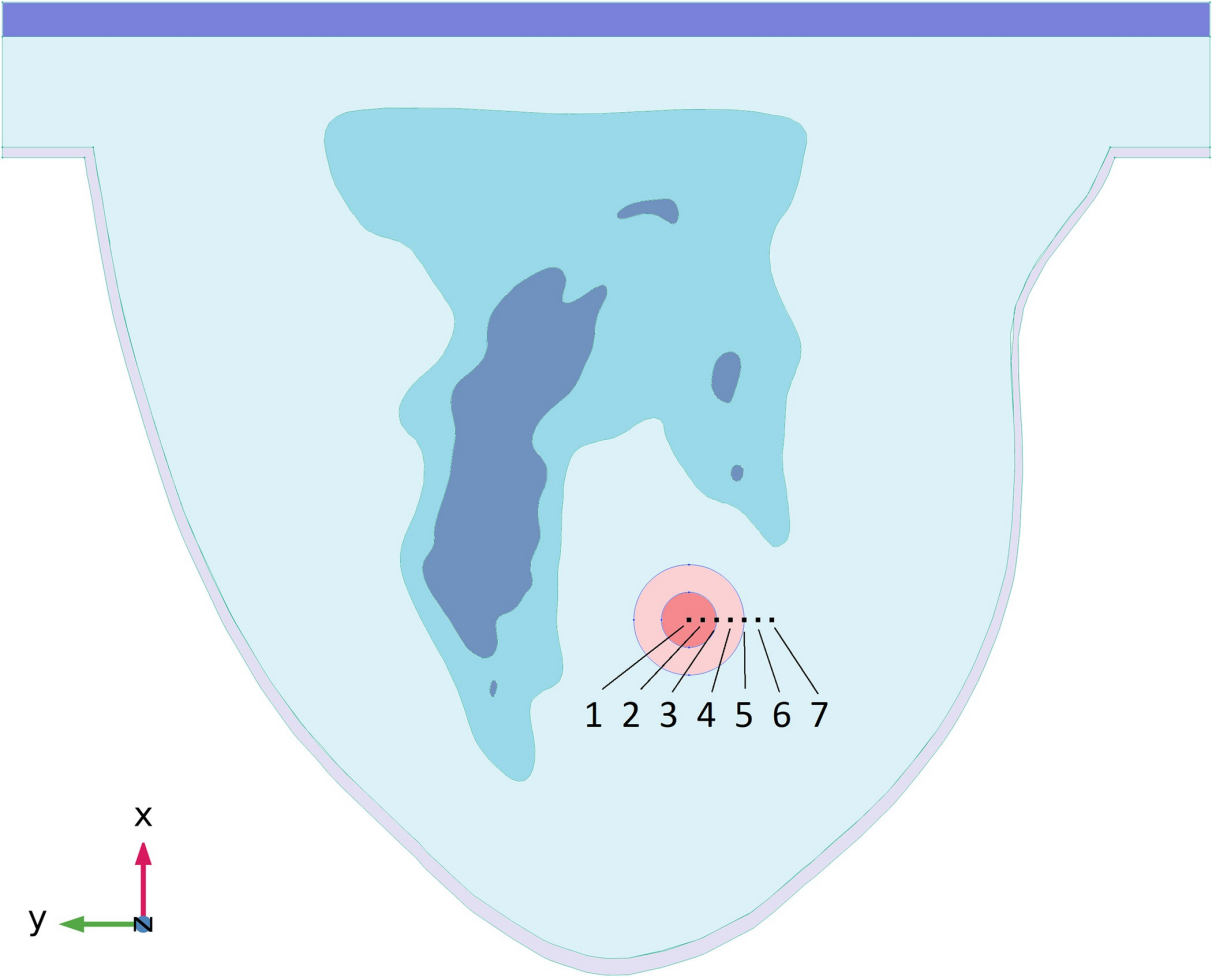
Position of observation points and their considered numbers on the cut surface 1.

Three sliced surfaces are evaluated to assess the simulation results, where the *x-y*, *x-z*, and *y-z* surfaces intersect the tumour’s centre. Fig 6 names and illustrates them. In addition, 7 observation points on cut surface 1 (shown in Fig 6) are considered for explaining results in various tumour areas. Each of these points has a 2 mm distance from the adjacent point. These points and their associated numbers are shown in Fig 7.

### 2.5 Mesh study

**Fig 8:**
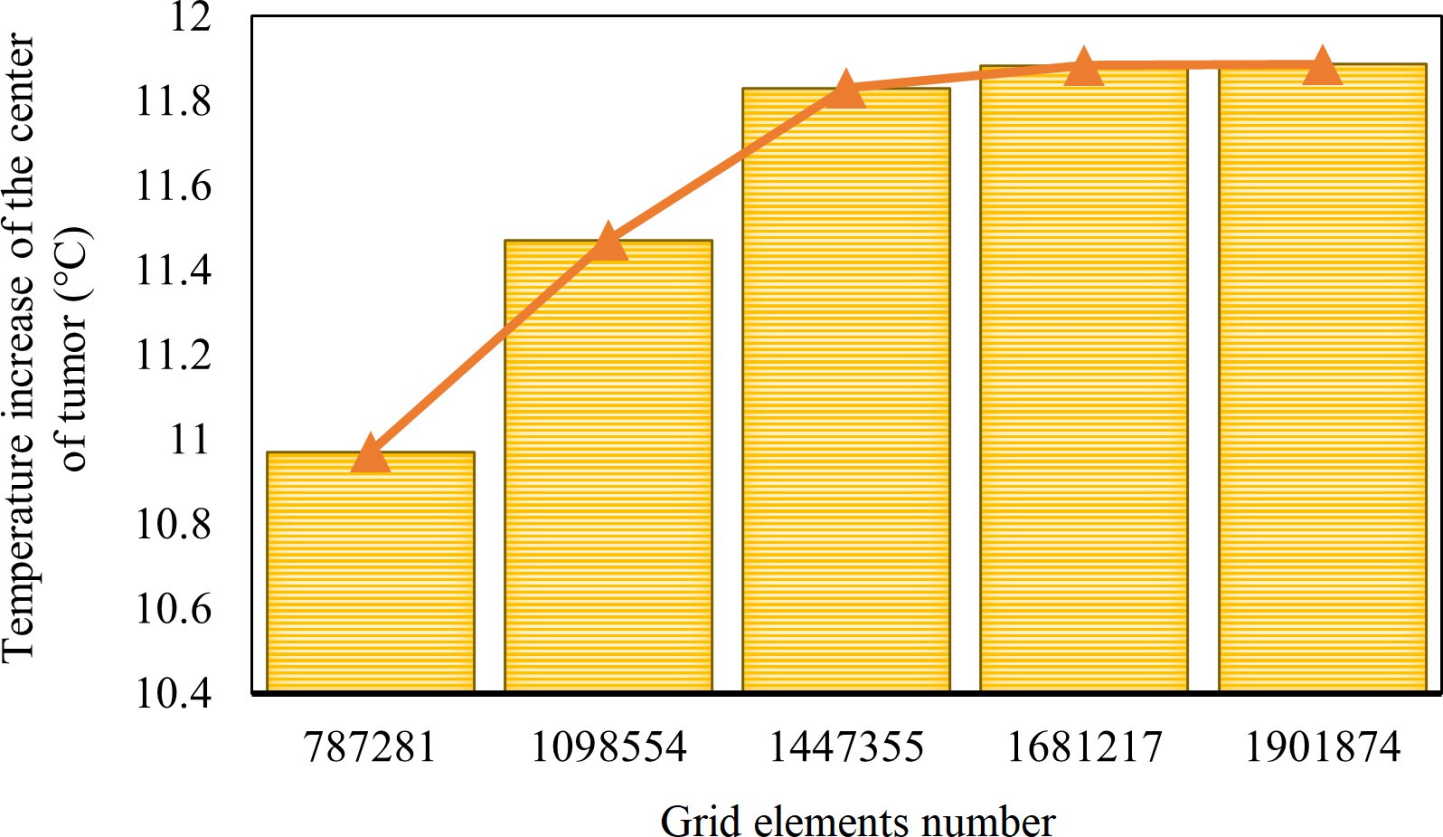
Grid independency test in the present study.

A few samples of the computational mesh are constructed, and the amount of temperature increase in the centre of the tumour is measured after 30 minutes of therapy under the magnetic field to study grid independency. A suitable number of grids is determined by balancing the expense of computational capabilities with the precision of simulation results. When the finer computational meshes do not affect the simulation results, the most refined grid is obtained to provide a reasonable grid independence level. Fig 8 shows the findings of this mesh study.

As shown in Fig 8, as the number of grids increases, the temperature rise in the tumour core approaches a nearly constant level. Table 2 shows the quantitative amounts and contrasts (errors that occur between them) of several meshed cases considered with the goal of determining the best case. The temperatures for cases 4 and 5 are substantially equal in Fig 8 and Table 2. Thus, case 4 is selected as the best option for reducing computational expenses.

**Table 2:** Quantitative amounts and relative errors of different cases.

Case	Grid Elements	Temperature increment (K)	% Relative error in comparison with the optimum case
**1**	787281	10.97014	−7.689%
**2**	1098554	11.47103	−3.474%
**3**	1447355	11.82971	−0.456%
**4**	1681217	11.88392	−
**5**	1901874	11.88671	+0.023%

**Fig 9:**
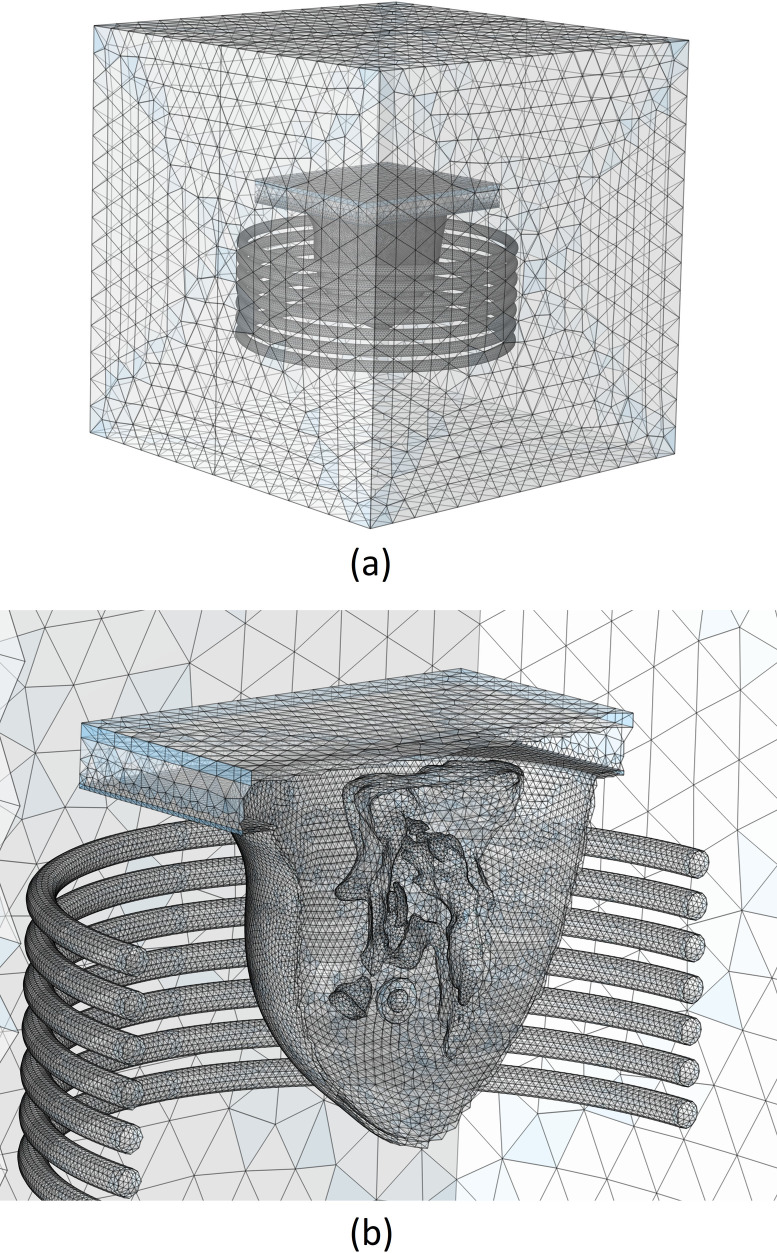
Optimum grid generation considered. (a) whole geometry, (b) sliced magnified model.

Fig 9(A) depicts the optimal mesh that was constructed. For a clearer presentation of the mesh grid and to observe the created mesh inside of the different tissues, the meshed model is sliced and illustrated in Fig 9(B).

### 2.6 Validation

In order to validate our method of simulation, first, the diffusion phenomenon (transport of diluted species physic) is validated with two different studies. Next, the heat induction phenomenon (magnetic field coupled with bioheat transfer physic) is validated by two different studies as well. These two validation steps are as follows:

#### 2.6.1 Diffusion phenomenon

For validating the diffusion of MNPs inside the tumor and breast, the diffusion of a spherical source that the diffusion substance is initially distributed uniformly through a sphere (like our simulations) is simulated in COMSOL and compared with the analytical results of Crank’s study [56] and also simulation results of Suleman et al.’s study [10]. Suleman et al. validated their own simulations with Crank’s investigation before. Thus, we validated our simulation with both of these results.

Based on Crank’s analytical study, the concentration *c* at radius *r* and time *t* for a spherical source of radius *a* with a uniformly initial concentration of *c*_0_ can be formalized as follows [56]:

$$c=\frac{1}{2}c_{0}\left\{erf\frac{a-r}{2\sqrt{Dt}}+erf\frac{a+r}{2\sqrt{Dt}}\right\}-\frac{c_{0}}{r}\sqrt{\frac{Dt}{\pi }[e^{\left(-{\left(a-r\right)}^{2}/4Dt\right)}-e^{\left(-{\left(a+r\right)}^{2}/4Dt\right)}]}$$ (29)

**Fig 10:**
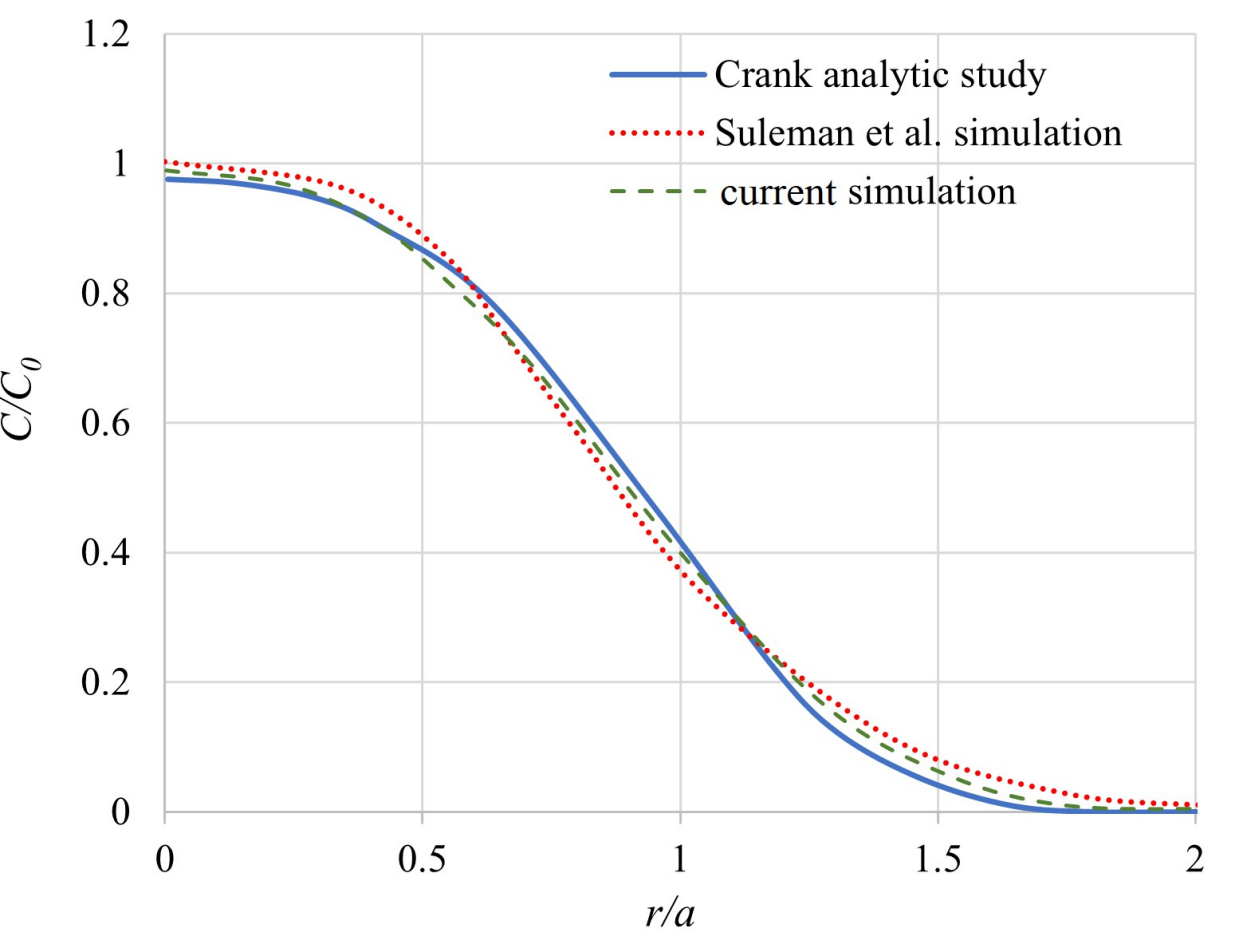
Comparison among current simulation, Suleman et al. simulation, and Crank analytic study plots of concentration distribution for a spherical source with uniformly initial concentration distribution.

In the above equation, *c*, *c*_0_, *a*, *r*, *D*, and *t* are concentration, the initial concentration of the source sphere, radius of the source sphere, radius, diffusion coefficient, and time respectively. The plot of concentration distribution for a spherical source and at (*Dt*/*a*^2^)^1/2^ = 1/4 from Crank’s study along with the same plots resulting from simulation by Suleman et al. and by us are shown in Fig 10 for comparison.

As can be seen, there are small gaps between our simulation, Suleman et al. simulation, and Crank’s analytical study results. These little variations can be regarded as tolerable inaccuracies.

#### 2.6.2 Heat induction phenomenon

To validate the simulation of heat induction inside tumour and breast tissues, we selected the Miaskowski et al. simulation results. Suleman et al. validate their simulation with their results, and we validate ours with both Miaskowski et al.’s simulation results and the results of Suleman et al.’s validation section.

**Fig 11:**
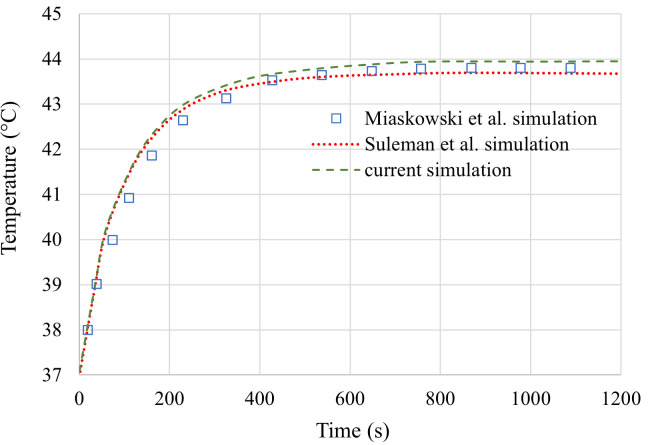
Comparison among current simulation, Suleman et al. simulation, and Miaskowski simulation plots of the maximum temperature in the breast model versus time.

Miaskowski et al. considered five concentric planar coils with a current equal to 400 A and frequency equal to 150 kHz to generate a magnetic field at the breast model area. They also considered that the MNPs only exist inside the tumour, and their concentration is uniform in every part of the tumour. An identical simulation model to Miaskowski et al. with the same frequency, coil arrangement, and coil winding number is considered for validation. So, a different model with a planar coil with a frequency of 150 kHz same as Miaskowski et al. is created for the validation. The point with the maximum temperature obtained in the whole breast model is considered and the plot of temperature difference of this point during 20 minutes of exposure is compared with Miaskowski et al. and Suleman et al.’s plots. It should be mentioned that in our simulation, the point with the maximum temperature obtained is the centre of the tumor. The plot of this comparison is shown in Fig 11.

As can be seen, the plot in the present study agrees well with the plots of Miaskowski et al. and Suleman et al. study. A relative error analysis is performed for a more quantitative comparison and is shown in Table 3. In this table, the maximum temperature difference obtained in the breast model after 1200 s for our simulation and Miaskowski et al. and Suleman et al.’s simulations is mentioned and compared. As demonstrated in Table 3, a satisfactory percentage rate of relative error has occurred between the acquired results and the simulation results by Miaskowski et al. and Suleman et al. Hence, our simulation method works well.

**Table 3:** Comparison between present simulation results and Miaskowski et al. and Suleman et al.’s simulation results [6, 10].

Maximum temperature difference in the breast model after 1200 s (°C)			% Relative error with Miaskowski’s result	% Relative error with Suleman’s result
Current study	Miaskowski’s result [6]	Suleman’s result [10]		
6.948	6.803	6.676	2.13%	4.07%

## 3. Results and discussion

This section will illustrate results acquired from different simulated physics in 4 different subsections. First, MNPs concentration distribution in tumours and breast tissue due to the diffusion phenomenon will be shown. Then, the magnetic field generated and the induced temperature distribution in the breast model will be shown. In the end, the distribution of necrotized tissue fraction will be presented, and the amount of tumour and breast tissues that dies due to the temperature rise will be evaluated.

### 3.1 Concentration distribution of MNPs

**Fig 12:**
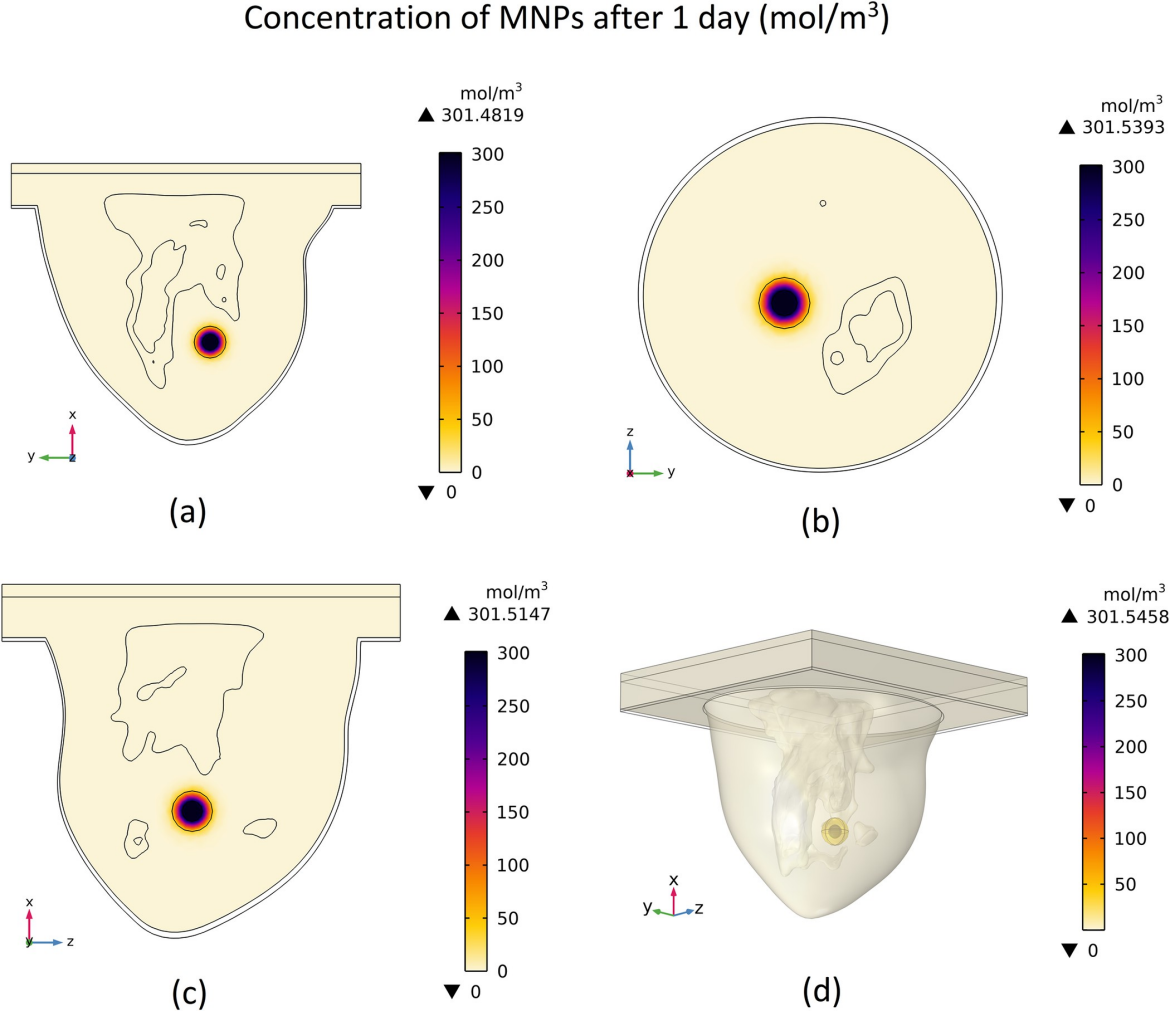
Distribution of MNPs’ concentration after 1 day of diffusion, (a) cut surface 1, (b) cut surface 2, (c) cut surface 3, (d) 3D view.

As mentioned before, after injection of the magnetic fluid in the centre of the tumour, total amount of magnetic fluid accumulates in a sphere with a radius equal to 4 mm. A duration of 1 day is given to the MNPs to diffuse completely inside the tumour and breast tissues. Thus, the concentration distribution of these MNPS inside the breast after one day is shown in Fig 12. The concentration contours for different mentioned cut surfaces along with the 3D view are shown in this figure.

**Fig 13:**
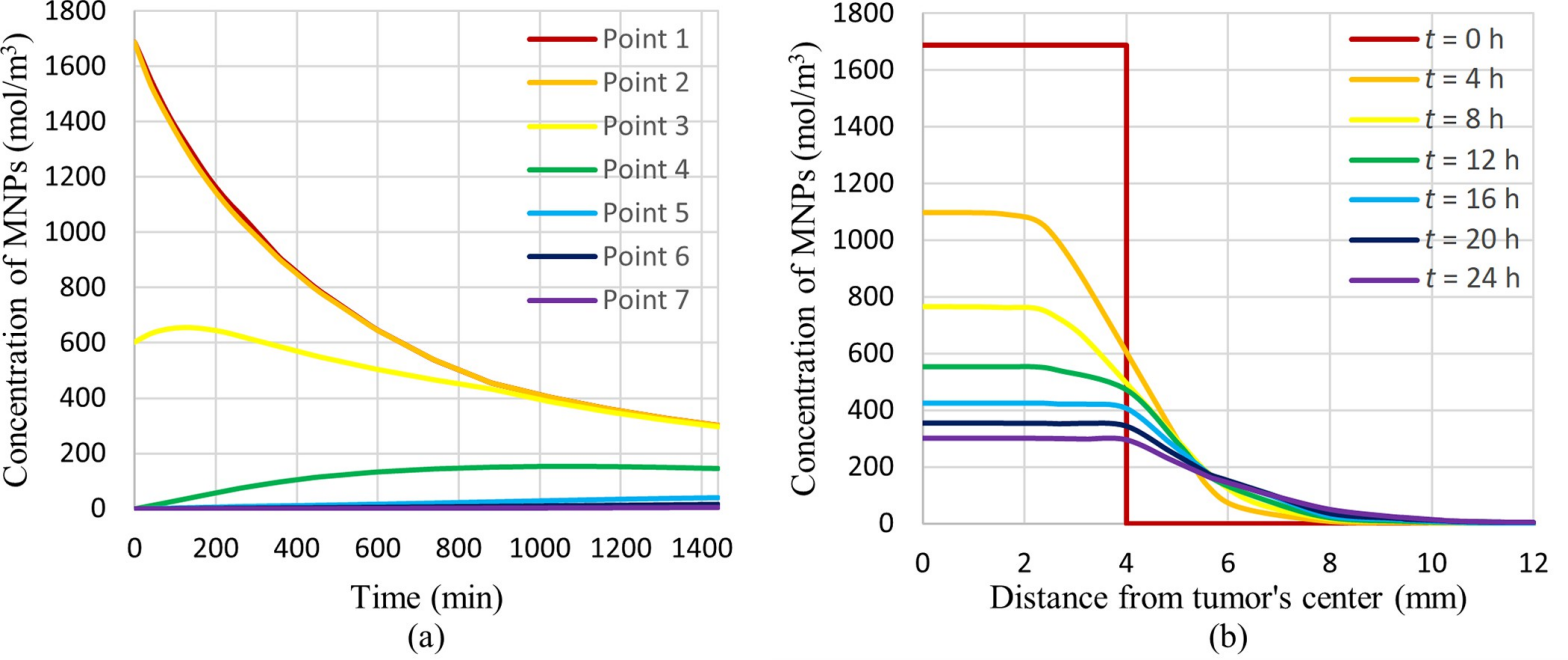
Concentration of MNPs versus, (a) time at different considered points, (b) distance from tumor’s center at different times.

Because of the existed concentration gradient, a net flow of MNPs will be created from a region of high concentration to a region of low concentration. This diffusion process results from the random motion of molecules. As can be seen in this figure, MNPs diffuse inside tumour tissue and, after that, a little inside the fatty breast tissue. The initial concentration of MNPs was considered to be 1669 mol m^-3^ in the sphere with a radius equal to 4 mm. After diffusion, the maximum concentration reached around 301.5 mol m^-3^ in the center of the tumor and gradually decreased by moving away from the center of the tumor. For a better illustration of the results relevant to the diffusion phenomenon, the plot of the MNPs concentration versus time at different specified points and also the plot of the concentration of MNPs versus distance from the tumour’s center at different times are shown in Fig 13.

As shown in Fig 13(A), due to the diffusion phenomenon, concentration in the centre or near centre points of the tumour decreases through time, and the concentration of points farther from the centre of the tumour rises through time. Also, in Fig 13(B) you can see the concentration distribution at different times. It can be seen that gradually MNPs are diffusing to the outer parts of the tumour and a little bit to the adjacent fatty tissue. After simulating the diffusion phenomenon, a magnetic field should be generated to induce heat in the tumour area. This generated magnetic field is shown in the following subsection.

### 3.2 Magnetic field generated

**Fig 14:**
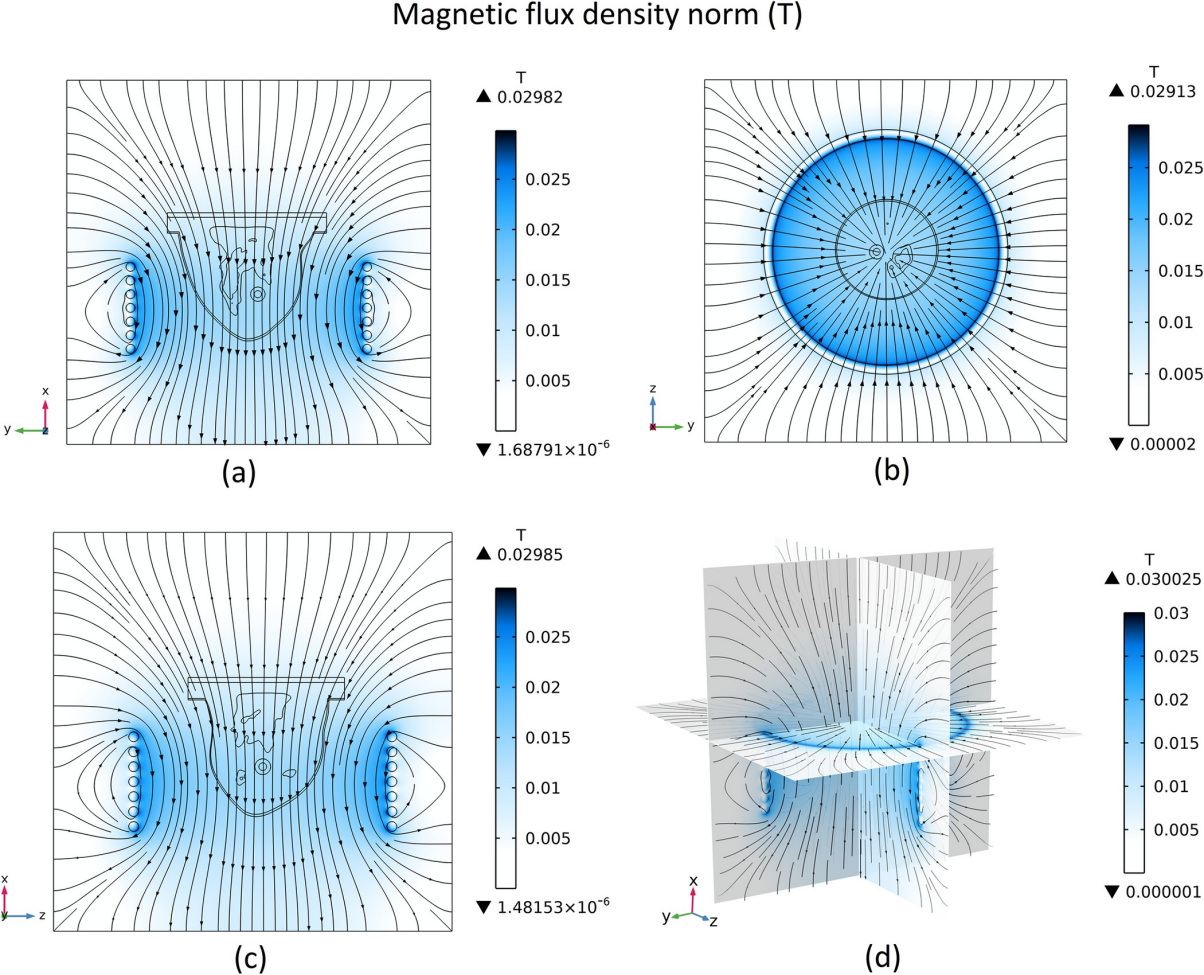
Magnetic flux density norm, (a) *x-y* surface, (b) *z-y* surface, (c) *x-z* surface, (d) 3D view.

Using seven circular copper coils mentioned that each coil carries a 400 A alternative current with a frequency equal to 400 kHz, generating a magnetic field in the breast area. The contour of the module of the magnetic flux density and the magnetic streamlines are shown in Fig 14. The contour and streamlines are shown for the three planes (*x-y*, *z-y*, and *x-z* surfaces) that pass through the middle of geometry (middle of the hypothetical cube).

The alternative current passing through the coils will create a changing electric field. This changing electric field will create an AC magnetic field at the location of the coils. Fig 14 depicts the generated magnetic field inside the coils. As can be seen, the module of the magnetic flux density is higher in areas near the coil and decreases by getting far from it. The maximum amount of the generated magnetic induction norm is around 0.03 T. This value is considered to be constant during the 30 minutes of exposure (therapy), and the temperature produced at the tumour area is obtained during this time.

There are several magnetic field exposure limits expressed by different researchers. For instance, Atkinson and Brezovich [57] proposed *H×f* = 4.85e+08 A m^−1^ s^−1^ as the safe exposure limit, where *H* and *f* are magnetic field intensity and frequency, respectively. Their test was based on the patient withstanding the treatment for more than one hour without any major discomfort. In our simulation, the maximum amount of magnetic field magnitude obtained in the tumor area is equal to 13.3 mT (10583.8 A m^-1^). So, the highest amount of *H×f* in the tumor area is equal to 4.23e+09 A m^−1^ s^−1^. This amount is higher than the Atkinson-Brezovich limit but still is lower than Hergt-Dutz [58], Kossatz et al. [59], and Mamiya [60] limits equal to 5e+09 A m^−1^ s^−1^, 8.3e+09 A m^−1^ s^−1^, and 18.7e+09 A m^−1^ s^−1^, respectively. Considering that our exposure time is 30 minutes and not 1 hour, this generated magnetic field can be considered safe. Different magnetic field intensities and frequencies can be opted by the clinician to reduce the feeling of discomfort for the patient.

### 3.3 Temperature distribution

**Fig 15:**
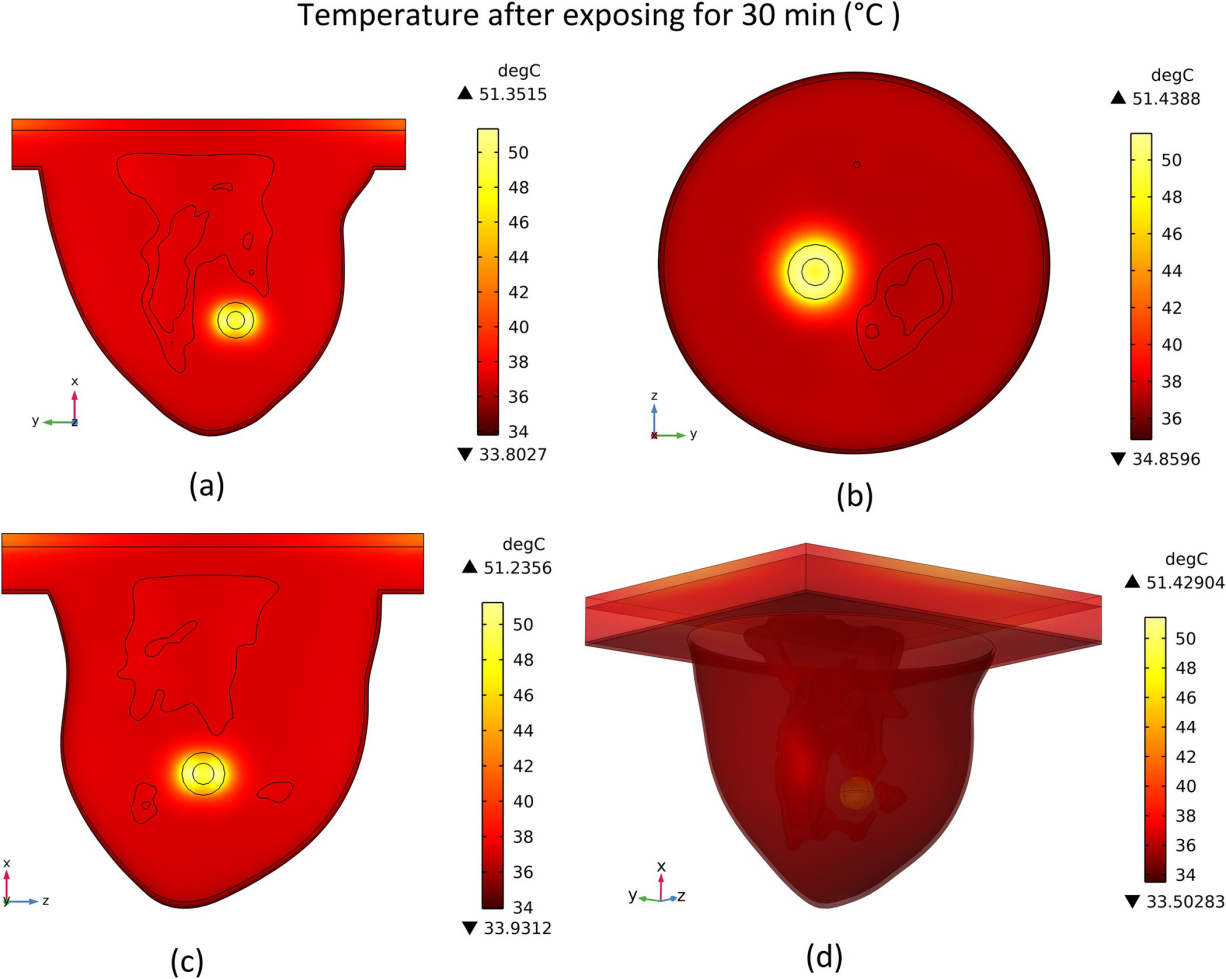
Distribution of temperature after 30-min-exposure to the magnetic field, (a) cut surface 1, (b) cut surface 2, (c) cut surface 3, (d) 3D view.

The conversion of heat from magnetic nanoparticles via magnetic energy loss will lead to a temperature increase at the location of MNPs. This heat generated will be transferred to other parts of the tumor and if the temperature rise is high enough it would kill the tissues. The produced temperature distribution after 30 minutes on different mentioned cut surfaces along with the 3D view is shown in Fig 15.

**Fig 16:**
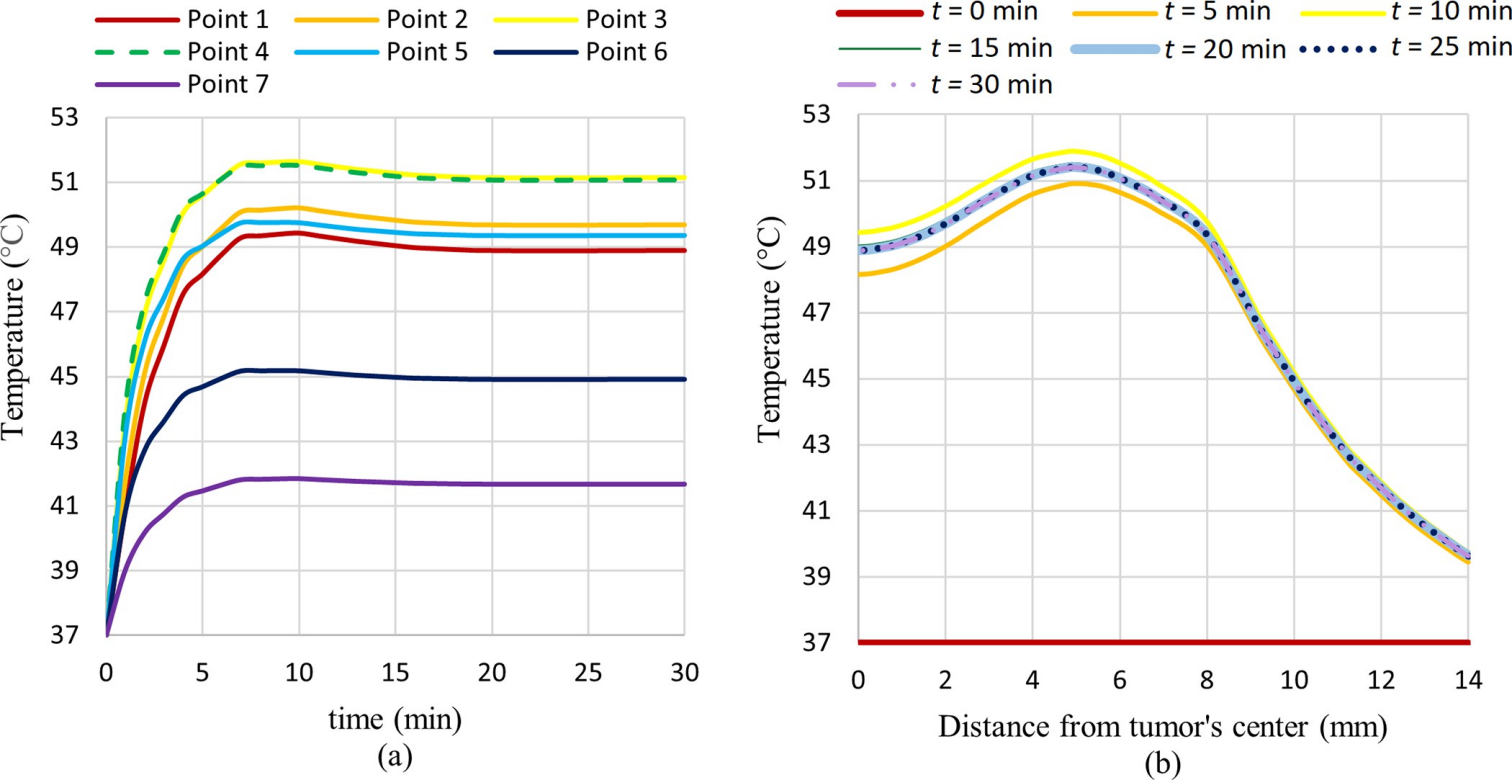
Temperature generated versus, (a) time at different considered points, (b) distance from tumor’s center at different times.

As can be seen in this figure, the temperature has risen in the tumor area and the maximum temperature achieved is about 51.4°C. This temperature increase will lead to necrosis and the death of tumor cells. For a better elaboration of the results, the plot of the temperature generated versus time at different specified points and also the plot of the temperature versus distance from the tumor’s center at different times are shown in Fig 16.

In Fig 16(A) and 16(B), it can be seen that temperature at different points rises to a maximum temperature due to the magnetic field excitation and, after that, reduces a little bit and stay constant for the rest of the exposure time. This little reduction in temperature is due to the blood perfusion and heat conduction to the outer areas of the tumour. The heat generation rate due to the magnetic field exposure is higher than this heat sink rate due to the blood perfusion and heat conduction, so it takes a little more time to reach the equilibrium temperature. This temperature fall after the initial heat increment is due to this phenomenon.

Also, it can be seen that the amount of temperature increase is higher at points near the tumour centre and will decrease by going farther from the tumour centre, which is rational due to the higher concentration of MNPs at the centre of the tumour. However, the interesting thing is that in point 3 (the point at 4 mm distance from the tumour’s centre), the temperature rise is higher than in point 1 (the point at the centre of the tumour). This phenomenon lies in the sinusoidal nature of the magnetic field. As shown in Fig 15, the temperature rise in two areas on the left and right sides of the tumour’s centre is higher than its centre. The magnetic field peak occurred in these areas and not precisely at the tumour’s centre. So, for comparison of temperature rise between points 1 and 3 that have approximately the same amount of MNPs concentrations (shown in Fig 13(B)), point 3 will reach higher temperatures because it is located at the magnetic field peak.

### 3.4 Distribution of necrotized tissue fraction

**Fig 17:**
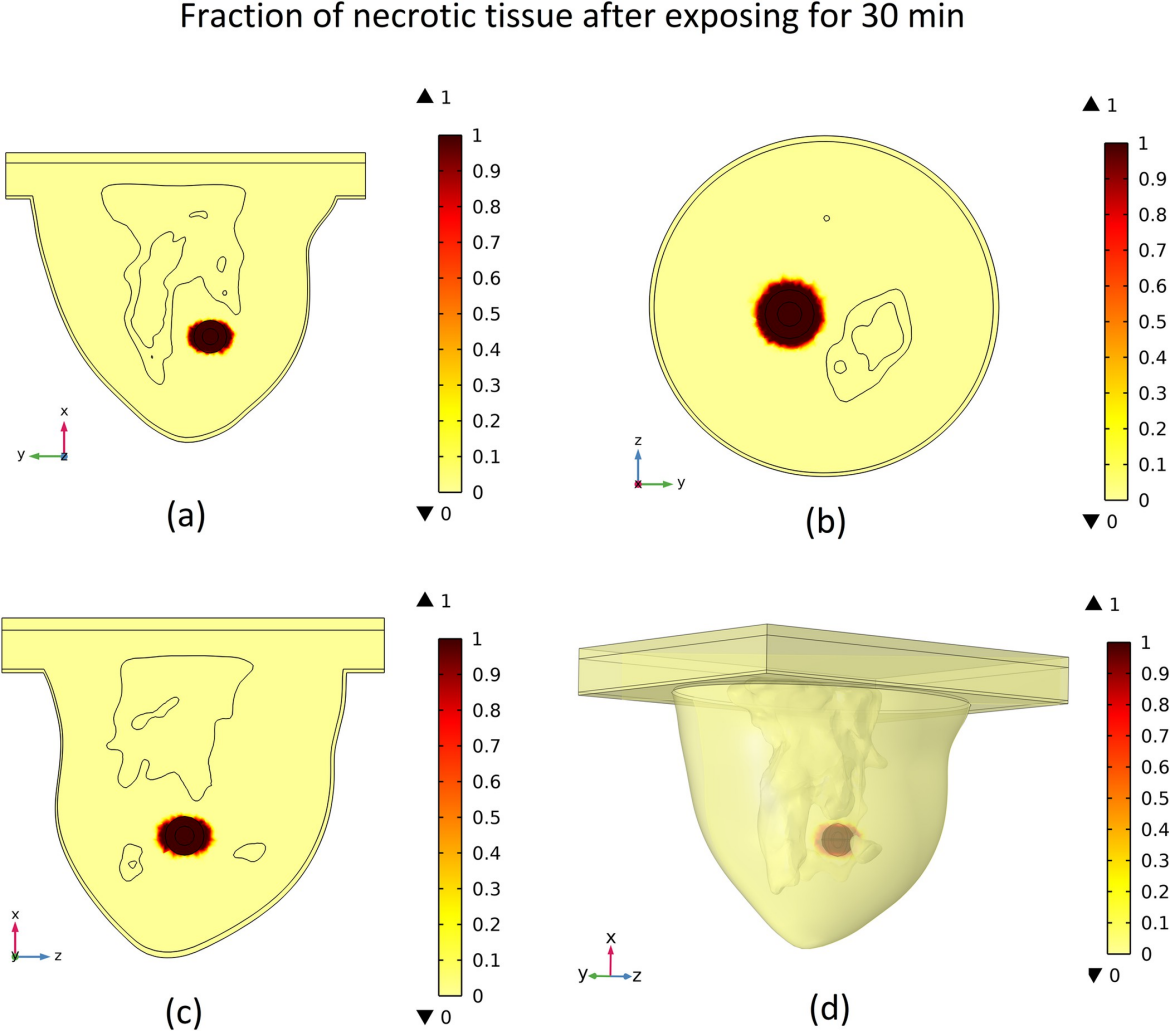
Distribution of fraction of necrotic tissue after 30 minutes of exposure to the magnetic field, (a) cut surface 1, (b) cut surface 2, (c) cut surface 3, (d) 3D view.

The temperature increase in the tumor area will lead to the ablation of tumor cells. When tissue temperature rises above a certain threshold, an irreversible cellular injury will occur, with interruption of metabolic processes. The distribution of the fraction of necrotic tissue after 30 minutes is shown in Fig 17. In this figure, the contour of the fraction of necrotic tissue on different mentioned cut surfaces along with the 3D view of the breast is illustrated.

**Fig 18:**
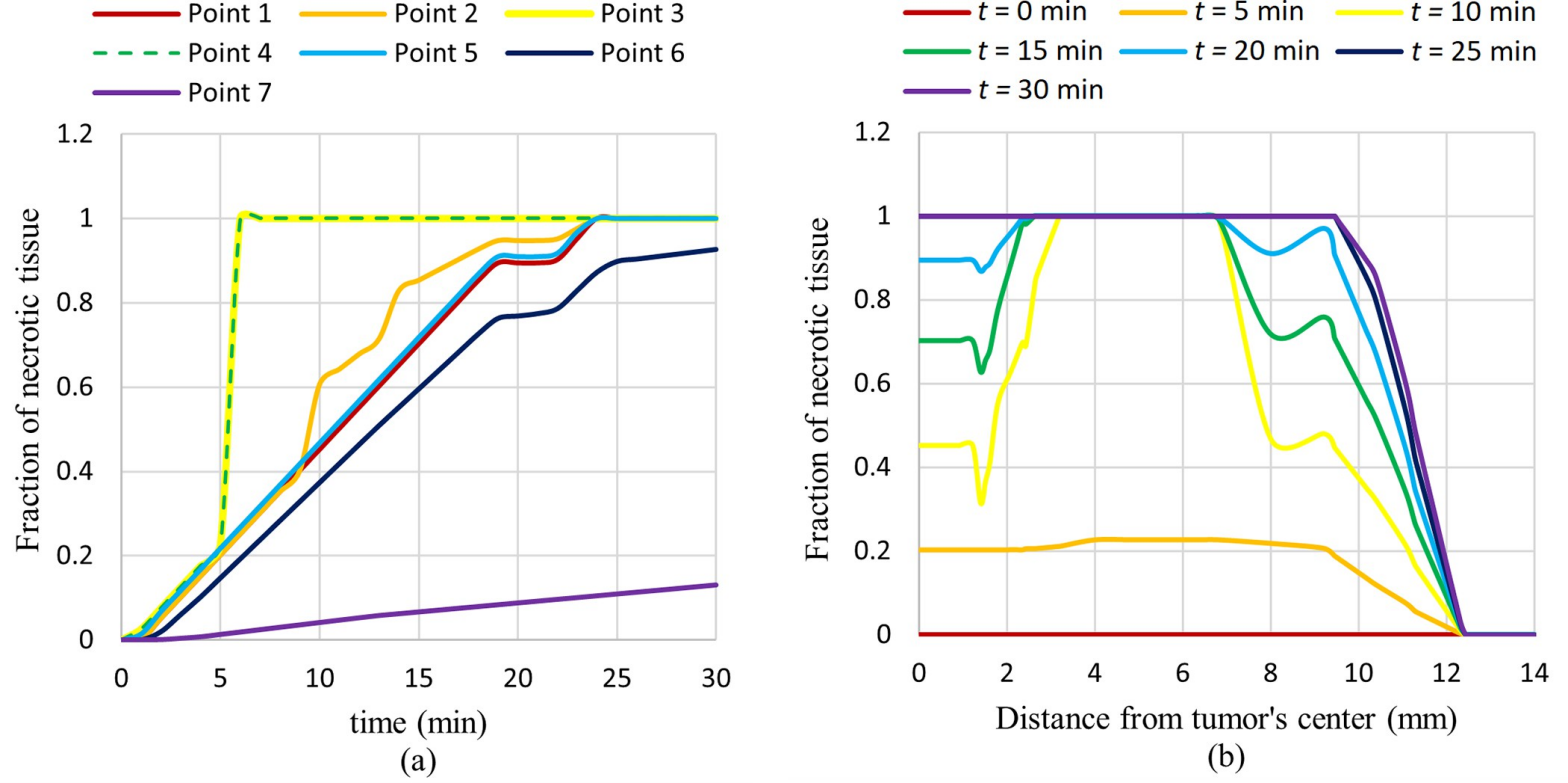
Fraction of necrotic tissue versus, (a) time at different considered points, (b) distance from tumor’s center at different times.

As shown in Fig 17, approximately all tumour tissues are ablated after 30 minutes of exposure, and a small portion of the normal tissues adjacent to the tumour has been destroyed. By comparing this figure with Fig 15, the relation between the amount of the necrotic tissue fraction and the temperature rise can be seen. Obviously, in areas with higher induced temperatures, the amount of tissue that will be ablated after 30 minutes of exposure will be higher. The plot of the fraction of necrotic tissue versus time at different specified points and also the plot of the fraction of necrotic tissue versus distance from the tumour’s centre at different times are shown in Fig 18.

As can be seen in this figure, the fraction of necrotized tissue will increase through the exposure time. This is rational because, through exposure time, tumour tissues will remain longer at high temperatures, leading to a higher proportion of cell damage. It can be seen that after 30 minutes of exposure, 100% of tumour tissues (points 1 to 5) are ablated, and some of the normal tissues adjacent to the tumour are ablated as well (points 6 and 7). Also, as said before, the fraction of necrotic tissue directly relates to the temperature. Thus, the fraction of necrotic tissue in areas with higher temperatures will be higher. This is the reason why a higher fraction of necrotic tissue will be obtained sooner in point 3, for example in comparison with point 1.

It should be mentioned that the overheating of adjacent tissues is unavoidable but can be reduced by changing different parameters such as the amount of nanofluid injected, frequency and amplitude of the magnetic field, and exposure time. This depends on the different MFH therapeutic procedures chosen by the clinician.

## 4. Conclusions

MFH can be considered one of the novel options available for ablation of deep-seated tumours like breast tumours, consisting of injection of magnetic nanofluid in the tumour area and exposure of the breast to an external magnetic field. This magnetic field will induce heat at the location of MNPs and lead to necrosis of tumour tissues. In the present investigation, the performance of a 3D mathematical modelling that can consistently solve the entire MFH phenomenon problem is examined. First, some of the outcomes of the present work are compared to other research outcomes cited in the article (to verify the simulation procedure). Afterward, an ARBP is utilized, and governing equations for all involving physics are solved numerically. MNPs concentration distribution, generated magnetic flux density, induced temperature distribution, and distribution of the necrotic tissue fraction are obtained in different steps for depicting the MFH phenomenon.

Results indicate that by injecting 0.1 mL diluted nanofluid consisting of magnetite MNPs with the base fluid water and using seven circular copper coils that each winding carries a 400 A alternative current with a frequency equal to 400 kHz, the temperature in tumour tissue can be raised to a maximum of about 51.4°C that leads to necrosis of entire tumour tissue after 30 minutes of EMF exposure.

The current simulation method can depict all the different physics involved in the MFH of the breast tumour in detail. This numerical platform can be used as an effective and reliable technique for predicting the outcome of any MFH process with different therapeutic conditions. various conditions may be applied based on each patient with specific tumour shapes and sizes (different levels of cancer development). These conditions are a higher or lower amount of magnetic nanofluid to be injected, a stronger or weaker magnetic field (higher or lower value of coil’s current or frequency) to induce higher or lower temperature in the tumour, and longer or shorter exposure time. This numerical platform can be utilized as an initial clinical tool for predicting and identifying the approximate results (the value of temperature rise and the fraction of tumour tissue cells that will be destroyed) for helping clinicians to choose the best therapeutic procedure based on different patients and different treatments that may be required. Also, this numerical platform can be used as a prediction tool to improve MFH experimental setups. By utilizing this platform, without any excessive expenses, it can be concluded that by changing each parameter in an MFH experimental setup how outcomes would be altered.

For achieving the current results, several physical factors have been simplified to solve the governing equations. This is one of the limitations of the discussed approach. Thus, this approach needs further modifications based on experimental results. For modifying this numerical simulation platform in order to obtain more accurate results, several *in-vivo* experiments should be performed and their results should be compared with the current numerical method’s results. The current numerical method should be modified accordingly. Therefore, as a future research direction, for the next step, several *in-vivo* and clinical experiments should be carried out. This numerical platform should be modified and improved based on the obtained results in order to be ready for utilization in clinical applications.

## Supporting information

S1 Data (XLSX)

## Abbreviations

**Nomenclature** A: frequency factor, s^-1^
*A*_coil_: total cross-section of the coil domain, m^2^
a: radius of spherical source, m
B: magnetic flux density, T
c: concentration, mol m^-3^
*c*_0_: initial concentration, mol m^-3^
*c*_p_: specific heat in constant pressure, J kg^-1^ K^-1^
*c*_p,b_: specific heat of blood, J kg^-1^ K^-1^
D: stokes-Einstein diffusion coefficient, m^2^ s^-1^
*D*_e_: electric flux density or electric displacement, C m^-2^
E: electric field strength, V m^-1^
$\vec{E}$: electric field vector, V m^-1^
$\vec{E_{s}}$: electric field on the surface vector, V m^-1^
f: electromagnetic field frequency, s^-1^
H: magnetic field strength, A m^-1^
$\vec{H}$: magnetic field vector, A m^-1^
*H*_0_: amplitude of magnetic field intensity, A m^-1^
h: convection heat transfer coefficient, W m^-2^ K^-1^
I: electric current, A
*I*_coil_: electric current of coil, A
J: current density, A m^-2^
*J*_coil_: current density of coil, A m^-2^
j: imaginary unit
K: magnetic anisotropy constant, J m^-3^
k: thermal conductivity, W m^-1^ K^-1^
k_B_: Boltzmann constant, J K^-1^
*M*_s_: magnetic fluid saturation magnetization, A m^-1^
N: number of coil turns
n: polynomial order of the Arrhenius equation
$\vec{n}$: surface normal vector
*P*_dis_: power dissipated by MNPs, W m^-3^
*Q*_met_: metabolic heat generation rate, W m^-3^
*Q*_MNPs_: heat dissipation rate by MNPs, W m^-3^
$\vec{q}$: heat transfer vector, J s^-1^
R: universal gas constant, J K^−1^ mol^−1^
r: radius, m
*r*_p_: radius of the MNPs, m
T: temperature, K
*T*_b_: arterial blood temperature, K
*T*_∞_: ambient temperature, K
t: time, s
VF: volume fraction of MNPs in the nanofluid
*V*_H_: hydrodynamic volume of the MNP, m^3^
*V*_M_: magnetic volume, m^3^
*V*_nf_: volume of nanofluid, m^3^
*V*_np_: total volume of nanoparticles, m^3^
*Greek symbols* α: degree of tissue injury
Δ*E*: activation energy, J mol^-1^
δ: surfactant layer thickness, m
ε: surface emissivity
ϵ_0_: permittivity of free space, F m^-1^
*ϵ*_r_: relative permittivity
η: viscosity, Pa s
θ: volume fraction of MNPs in tissue
*θ*_d_: fraction of necrotic tissue
μ_0_: permeability of free space, H m^-1^
*μ*_r_: relative permeability
ρ: density, kg m^-3^
*ρ*_b_: density of blood, kg m^-3^
σ: electrical conductivity, S m^-1^
σ_SB_: Stefan-Boltzmann constant, W m^-2^ K^-4^
τ: relaxation time, s
τ_B_: Brownian relaxation time, s
*τ*_N_: Neel relaxation time, s
*χ*_0_: ferrofluid susceptibility
${\vec{\varphi }}_{i}$: diffusive flux vector of i component, mol m^-2^ s^-1^
ω: angular frequency, rad s^−1^
*ω*_b_: blood perfusion rate, s^-1^
